# Determination and Quantification of Phytochemicals from the Leaf Extract of *Parthenium hysterophorus* L. and Their Physio-Biochemical Responses to Several Crop and Weed Species

**DOI:** 10.3390/plants11233209

**Published:** 2022-11-23

**Authors:** HM Khairul Bashar, Abdul Shukor Juraimi, Muhammad Saiful Ahmad-Hamdani, Md. Kamal Uddin, Norhayu Asib, Md. Parvez Anwar, Ferdoushi Rahaman, SM Rezaul Karim, Mohammad Amdadul Haque, Zulkarami Berahim, Nik Amelia Nik Mustapha, Akbar Hossain

**Affiliations:** 1 Department of Crop Science, Faculty of Agriculture, University Putra Malaysia, Serdang 43400, Malaysia; 2 Bangladesh Agricultural Research Institute (BARI), Gazipur 1701, Bangladesh; 3 Department of Land Management, University Putra Malaysia, Serdang 43400, Malaysia; 4 Department of Plant Protection, Faculty of Agriculture, University of Putra Malaysia, Serdang 43400, Malaysia; 5 Department of Agronomy, Faculty of Agriculture, Bangladesh Agricultural University, Mymensingh 2202, Bangladesh; 6 Laboratory of Climate-Food Crop Production, Institute of Tropical Agriculture and Food Security, Universiti Putra Malaysia, Serdang 43400, Malaysia; 7 Department of Agronomy, Bangladesh Wheat and Maize Research Institute, Dinajpur 5200, Bangladesh

**Keywords:** parthenium weed, bioherbicides, chlorophyll content, photosynthetic rate, stomatal conductance, enzymatic activity, physio-biochemical responses

## Abstract

This current investigation was undertaken both in laboratory and glasshouse for documentation and quantification of phytochemicals from different parts of the parthenium (*Parthenium hysterophorus* L.) plant through LC-MS and HPLC to study their effect on two crops namely, Bambara groundnut (*Vigna subterranean* L.) and maize (*Zea mays* L.), and six different types of weed e.g., *Digitaria sanguinalis*, *Eleusine indica*, *Ageratum conyzoides*, *Cyperus iria*, *Euphorbia hirta*, and *Cyperus difformis*. The parthenium methanolic leaf extracts at 25, 50, 75, and 100 g L^−1^ were sprayed in the test crops and weeds to assess their physiological and biochemical reactions after 6, 24, 48, and 72 h of spraying these compounds (HAS). The LC-MS analysis confirmed seven types of phytochemicals (caffeic acid, ferulic acid, vanillic acid, parthenin, chlorogenic acid, quinic acid, and p-anisic acid) in the parthenium leaf extract that were responsible for the inhibition of tested crops and weeds. From the HPLC analysis, higher amounts in leaf methanol extracts (40,752.52 ppm) than those of the stem (2664.09 ppm) and flower extracts (30,454.33 ppm) were recorded. Parthenium leaf extract at 100 g L^−1^ had observed higher phytotoxicity on all weed species except *C. difformis*. However, all crops were found safe under this dose of extraction. Although both crops were also affected to some extent, they could recover from the stress after a few days. The photosynthetic rate, transpiration rate, stomatal conductance, carotenoid and chlorophyll content were decreased due to the application of parthenium leaf extract. However, when parthenium leaf extract was applied at 100 g L^−1^ for 72 h, the malondialdehyde (MDA) and proline content were increased in all weeds. Enzymatic antioxidant activity (e.g., superoxide dismutase (SOD), catalase (CAT), and peroxidase (POD) contents) were also elevated as a result of the sprayed parthenium leaf extract. The negative impact of physiological and biochemical responses as a consequence of the parthenium leaf extract led the weed species to be stressed and finally killed. The current findings show the feasibility of developing bioherbicide from the methanolic extract of parthenium leaf for controlling weeds, which will be cost-effective, sustainable, and environment friendly for crop production during the future changing climate.

## 1. Introduction

Parthenium (*Parthenium hysterophorus* L.) is one of the most invasive weeds on the planet, endangering natural ecosystems and agroecosystems in more than 30 countries. It causes crop and pasture losses, degrades natural plant biodiversity, poses human and animal health risks, and causes substantial economic losses to people and their interests [1]. Controlling this noxious weed is difficult due to its high germination rate, prolific nature, and resilience to chemical herbicides. However, the utilization of this invasive species for the production of a value-added product is another avenue of parthenium management. Allelopathy is a biological phenomenon that occurs when living species in an ecosystem interact chemically and affect the growth of neighboring plants [2]. Several previous studies indicated that parthenium possesses an allelopathic potential that could be used in weed control [3].

Therefore, a method for developing a bioherbicide might be the identification and separation of the allelopathic chemicals from *P. hysterophorus.* The allelopathic property of parthenium plants has been linked to terpenoids, steroids, phenols, coumarins, flavonoids, tannins, alkaloids, and cyanogenic glycosides, as well as their breakdown products [3]. In terms of phytotoxicity, phenolic compounds have been the subject of the greatest investigation among these substances. These substances are biologically active because they inhibit weed seed germination and seedling growth [4]. The main allelochemicals in parthenium were found to be *p*-coumaric, *p*-hydroxybenzoic, ferulic acid, and vanillic acid in the phenolic compounds [5]. 

Weeds are unwanted plants in crop fields, which play a significant role in yield losses [6]. In rainfed agricultural systems, crops and weeds grow together and compete for light, space, nutrients, and water [7]. Diversified weed species are found to grow in crop fields, among which *Cyperus iria* (L.)*, Ageratum conyzoides* (L.)*, Digitaria sanguinalis* (L.)*, Eleusine indica* (L.)*, Cyperus difformis* (L.)*,* and *Euphorbia hirta* (L.) are major weeds in cultivated fields. *Agetarium conyzoides* is an invasive weed that has infected cultivated regions and hampered agricultural growth [8]. *Cyperus iria* is a tufted, tall, and invasive annual herbaceous sedge that substantially reduces crop yields [9,10]. *Digitaria sanguinalis* is a common weed in nonirrigated farmlands that infest cultivated areas and lowers crop output, especially in wheat, maize, and soybean fields [11]. *Eleusine indica* is one of the world’s top ten worst weeds that causes major productivity losses in Malaysian vegetable and fruit crops [12]. In rice fields, *C. difformis* is a malignant weed that poses a danger to rice production [13].

Agriculture faces a difficult problem when trying to manage weeds in crop fields [14]. Owing to their greater effectiveness, lower cost, and quicker payback, farmers primarily prefer chemical herbicides to manage weeds. Another big issue with reliance in some countries is the movement of labor away from agriculture to industries or other nations for jobs [14]. However, overuse of synthetic herbicides may increase the number of herbicide-resistant biotypes [15], and decrease agricultural output, environmental pollution, and health risks [16]. On the other hand, the use of bioherbicides developed from allelochemicals or allelopathic plants can play a significant role as a replacement for the reliance on synthetic chemical herbicides in sustainable agriculture [17].

Herbicidal phytotoxicity on weed growth is caused by a reduction in root cell division, food absorption, growth hormone development, and pigment formation, as well as the production of reactive oxygen species (ROS), stress-related hormones, and aberrant antioxidant activity [18]. Herbicide exposure can cause a variety of physiological and biochemical reactions in plants, including chlorosis, lipid peroxidation (LPO), and antioxidant responses [19,20]. Lipid peroxidation has an impact on the cell’s physiological processes. Malondialdehyde (MDA), a lipid peroxidation metabolic product, is a widely recognized biomarker of oxidative stress in plants [21]. The presence of an antioxidant system in plants is thought to be responsible for herbicide tolerance [22]. Ascorbic acid (vitamin C), α-tocopherol, glutathione, and carotenoids are among the nonenzymatic components, whereas superoxide dismutase (SOD), catalase (CAT), peroxidase (POX), ascorbate peroxidase (APX), glutathione reductase (GR), glutathione S-transferase (GST), etc., are common enzymatic components in plants [23]. The superoxide radical is scavenged by SOD, whereas hydrogen peroxide (H_2_O_2_) is scavenged by CAT and APX [24]. Various abiotic conditions cause plants to produce excessive amounts of ROS, which are extremely reactive and poisonous and cause damage to proteins, lipids, carbohydrates, and DNA, resulting in oxidative stress [25]. By scavenging ROS and reducing LPO, carotenoids serve an important photo-protective role in plants [25]. Furthermore, chlorophylls are the primary pigments involved in photosynthesis, and changes in plant leaf chlorophyll concentration indicate resistance and photosynthetic abilities [19,26,27,28]. 

A good number of studies on the allelochemicals of parthenium weed and their effect on other plants and weeds are available, but studies on quantification and physio-biochemical (mode of action) responses of parthenium allelochemicals are limited. In the context, the current study was undertaken for development of bioherbicides from leaf, stem, and flower extracts of *P. hysterophorus*. The ultimate goal of this study is to determine and quantify phytochemicals from different parts of parthenium through LC-MS and HPLC analysis; lead to find out the best biochemical for using as bioherbicides as a cost-effective and environmentally friendly approach for controlling weeds in crop fields. 

## 2. Results

### 2.1. Identified Phytochemicals from P. hysterophorus through LC-MS Analysis

**Figure 1:**
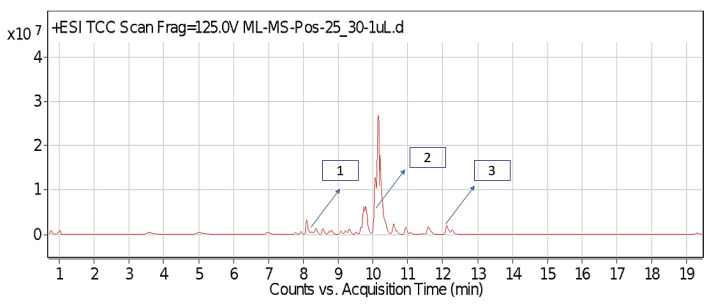
LC-MS chromatograms showing the phytotoxic compound positive ion mode of *P. hysterophorus* leaf methanolic extract (1. Chlorogenic acid, 2. Parthenin, and 3. Quinic acid).

**Figure 2:**
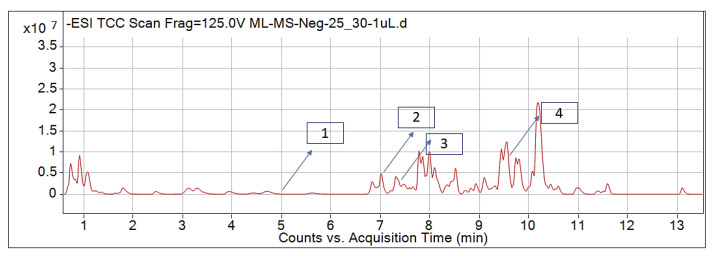
LC-MS chromatograms showing the phytotoxic compounds negative ion mode of *P. hysterophorus* leaf methanolic extract (1. P-Anisic acid, 2. Caffeic acid, 3. Vanillic acid, and 4. Ferulic acid).

The leaf, stem, and flower extracts of *P. hysterophorus* have diverse chemical compositions. A total of seven phenolic derivatives were detected from the methanol extract of *P. hysterophorus* in different parts through LC-MS analysis (Table 1). These phenolic derivatives are responsible for the inhibition of other plants, autotoxic, and dermatitis. Parthenin and other phenolic acids found in the leaf and flower extracts include vanillic acid, caffeic acid, quinic acid, anisic acid, chlorogenic acid, and ferulic acid; contrary parthenin, vanillic acid were found in the stem extract. For most of the compounds, [M-H]^+^ and [M-H]^−^ ions were observed. The total ion current chromatography in positive and negative ESI mode is shown in Figure 1 and Figure 2. Quinic acid, parthenin, and chlorogenic acid were identified by positive ionization mode at 12.116, 10.004, and 8.09 min, with 181.12, 263.1267, and 300.183 m/z, respectively. Another four phenolics, caffeic acid, ferulic acid, vanillic acid, and p-anisic acid, were documented from negative polarity analysis at 7.183, 9.84, 7.367, and 5.121 min with m/z 341.0894, 193.05129, 153.01983, and 151.04047.

### 2.2. Quantification of Phytochemicals from P. hysterophorus through HPLC Analysis

Based on the results obtained in the bioassays, the bioactive extracts of *P. hysterophorus* aerial parts were evaluated by HPLC analysis and performed to quantify the secondary metabolites. The number of identified compounds and their amounts (expressed as ppm) depended on the plant parts (Table 2). 

The quantification of identified compounds was performed using regression equations. All the identified compounds were detected at higher amounts in leaf methanol extracts than in those of stem and flower extracts. 

Quinic acid was found to be the most abundant component in leaf methanol extract (36,504.48 ppm), followed by parthenin (4208.08 ppm) and then chlorogenic acid (17.841 ppm). Again, from flower methanol extracts, the highest amount detected was quinic acid (26,528.56 ppm), followed by parthenin (3823.67 ppm) and chlorogenic acid (65.270 ppm). From the above discussion, it is clear that quinic acid was in the highest amount in leaf methanol extract (36,504.48 ppm), followed by flower methanol extract (26,528.56 ppm). On the other hand, very small quantities of these chemicals were present in the stem extract, containing parthenin and vanillic acid only.

### 2.3. Effect on Chlorophyll-ɑ Content of the Crops and Weeds

The chlorophyll-*ɑ* content of Bambara groundnut, maize, *D. sanguinalis, E. indica*, *A*. *conyzoides, C. iria, E. hirta,* and *C. difformis* was significantly affected by foliar sprays of *P. hysterophorus* leaf methanol extract at 6, 24, 48, and 72 hrs after spray (HAS) (Table 3). 

**Figure 3:**
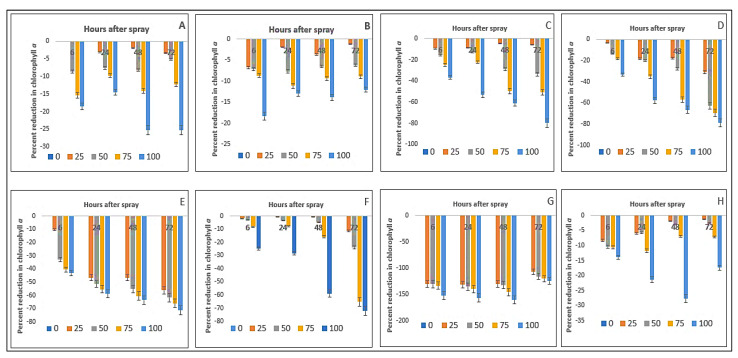
Reduction (%) of chlorophyll-*a* as a consequence of parthenium leaf methanol extracts on several crops and weeds: (**A**)—Bambara groundnut; (**B**)—Maize; (**C**)—*D. sanguinalis*; (**D**)—*E. indica*; (**E**)—*A. conyzoides*; (**F**)—*C. iria*; (**G**)—*E. hirta*, and (**H**)—*C. difformis*.

Chlorophyll-*ɑ* content showed a decline from the highest concentration (100 g L^−1^), and 48 to 72 h after spray. In the case of *E. hirta,* the greatest decline was observed at 48 HAS (160.41%), followed by 79.84% in *D. sanguinalis*, 78.85% in *E. indica*, 71.22% in *A. conyzoides*, and 72% in *C. iria* at 72 HAS. On the other hand, the decline was 27.67% in *C. difformis*, 13.82% in maize, and 25.32% in Bambara groundnut at 48 HAS. Among the weed’s chlorophyll-*ɑ* contents, the greatest decline was in *E. hirta* (160.41%) and the lowest was in *C. difformis* (27.67%) (Figure 3). It was observed here that maize and Bambara groundnut crops showed less decline in chlorophyll-*ɑ* content and among the weeds, *C. difformis* showed less decline in chlorophyll content. On the other hand, although the crops were affected to some extent in their chlorophyll-*ɑ* content due to the application of parthenium extract, these were recovered later and confirmed by the deep green appearance of the crop leaves.

### 2.4. Effect on Chlorophyll-ɓ Content of the Crops and Weeds

**Figure 4:**
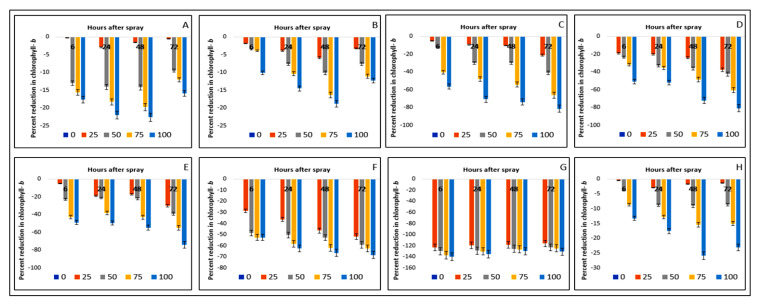
Reduction (%) of chlorophyll-*b* as a consequence of parthenium leaf methanol extract on several crops and weeds: (**A**)—Bambara groundnut; (**B**)—Maize; (**C**)—*D. sanguinalis*; (**D**)—*E. indica*; (**E**)—*A. conyzoides*; (**F**)—*C. iria*; (**G**)—*E. hirta*, and (**H**)—*C. difformis*.

The chlorophyll-*ɓ* contents of the crops and weeds were also affected by the foliar sprays of parthenium extract (Table 3). A similar trend of reduction in chlorophyll-*ɓ* contents and response to the parthenium extract application was noticed in the crop species. Interestingly, a higher reduction in chlorophyll-*ɓ* content was noticed in the weed species; e.g., 81.55% in *D. sanguinalis*, 80.91% in *E. indica*, 74% in *A. conyzoides*, 68.33% in *C. iria*, 130.94% in *E. hirta,* and 25.87% in *C. difformis* reduction occurred when plants were applied with 100 g L^−1^ and measured at 72 HAS (Figure 4). 

Among the weed species, the highest decline again was found in *E. hirta* (130.94%) followed by *D. sanguinalis* (81.55%) and *E. indica* (80.91%) (Figure 4). The results also exhibited that the decline in chlorophyll-*ɓ* content was linearly related to the concentration level of extract and hours after spraying for all weeds. This produced a greater impact on the green pigment and thereby on the photosynthesis of the plants.

### 2.5. Effect on Total Chlorophyll Content of the Crops and Weeds

**Figure 5:**
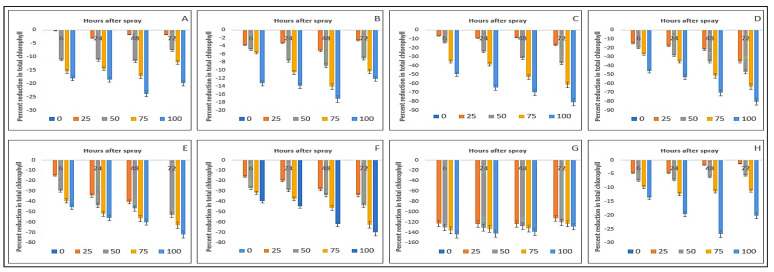
Reduction (%) of total chlorophyll as a consequence of parthenium leaf methanolic extracts on several crops and weeds: (**A**)—Bambara groundnut; (**B**)—Maize; (**C**)—*D. sanguinalis*; (**D**)—*E. indica*; (**E**)—*A. conyzoides*; (**F**)—*C. iria*; (**G**)—*E. hirta*, and (**H**)—*C. difformis*.

**Figure 6:**
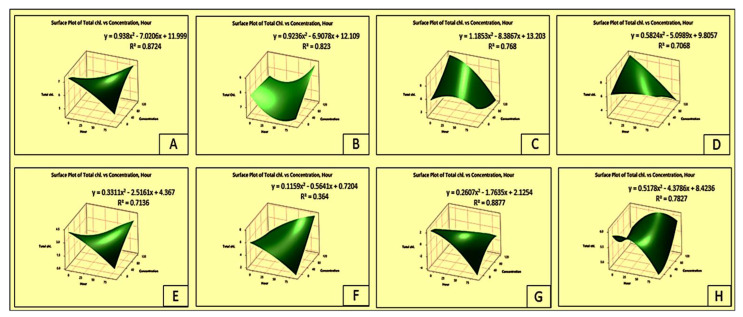
Surface plot of total chlorophyll vs. concentration hours as a consequence of parthenium leaf methanol extract on several crops and weeds: (**A**)—Bambara groundnut; (**B**)—Maize; (**C**)—*D. sanguinalis*; (**D**)—*E. indica*; (**E**)—*A. conyzoides*; (**F**)—*C. iria*; (**G**)—*E. hirta*, and (**H**)—*C. difformis*.

The effects of parthenium extracts on the chlorophyll-*a* and chlorophyll-*ɓ* contents of the test plants are depicted in the total chlorophyll content of the crops and weeds (Table 4). Accordingly, the crop species were less affected, but the weed species were greatly affected by the foliar sprays of parthenium extracts. The trend of reduction in chlorophyll-*a* and chlorophyll-*ɓ* contents of the test species was similar (Figure 5 and Figure 6). 

Figure 6 and Tables S1–S5 display the total chlorophyll response surface plot as a consequence of parthenium leaf methanol extract on Bambara groundnut, maize, *D. sanguinalis, E. indica, A. conyzoides, C. iria, E. hirta, and C. difformis*. The chart shows that as the concentration increased, the total chlorophyll content gradually declined. However, 72 HAS saw a recovery in the total chlorophyll contents in Bambara groundnut, maize, and *C. difformis* (Figure 6 and Tables S1–S5).

### 2.6. Effect on Carotenoid Content of the Crops and Weeds

**Figure 7:**
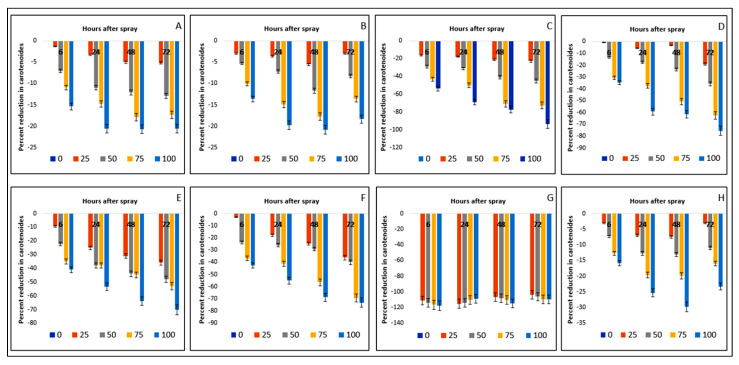
Reduction (%) of carotenoids due to the effect of parthenium leaf methanol extract on several crops and weeds: (**A**)—Bambara groundnut; (**B**)—Maize; (**C**)—*D. sanguinalis*; (**D**)—*E. indica*; (**E**)—*A. conyzoides*; (**F**)—*C. iria*; (**G**)—*E. hirta*, and (**H**)—*C. difformis*.

Carotenoid contents of crops and weed species were reduced by parthenium leaf methanol extract foliar spray at different exposure times (Table 4). The reduction in carotenoid content was also positively related to the concentration of extract and time of exposure. Bambara groundnut was found to lose carotenoid by 20.71% and maize by 20.85% at 48 HAS, compared to the control. All the weed species were greatly affected by the foliar sprays of parthenium extract and the trend of reduction was as follows: 93.60% in *D. sanguinalis*, 75.39% in *E. indica*, 70.08% in *A. conyzoides*, 73.12% in *C. iria*, and 29.81% in *C. difformis*, observed at 72 HAS. Worth noting is that *E. hirta* lost its carotenoid by 117.72%, even at 6 HAS (Figure 7). The trend of reduction in the weed species was similar to the reduction in chlorophyll contents.

### 2.7. Effect on Photosynthesis Rate of Crops and Weeds

The photosynthetic rates of both the crop and weed species were reduced due to foliar sprays of parthenium extract. Of the two crops, Bambara groundnut was more affected than maize, although all the weed species were affected in their photosynthetic rates with the impact of time after spray and extract concentration in the weed species (Table 5).

**Figure 8:**
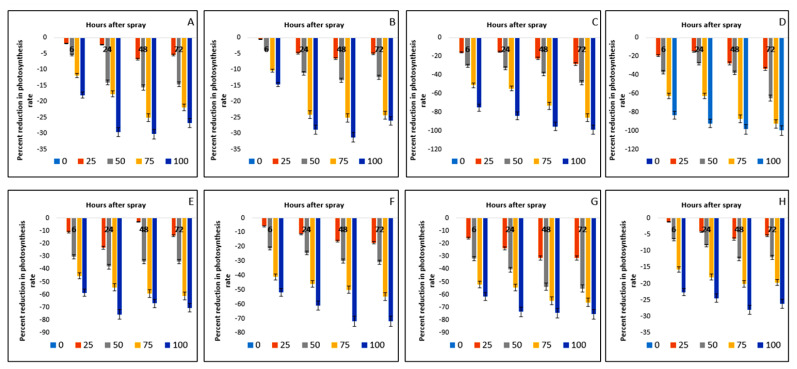
Reduction (%) of photosynthesis rate of several crops and weeds due to the effect of parthenium leaf methanol extract: (**A**)—Bambara groundnut; (**B**)—Maize; (**C**)—*D. sanguinalis*; (**D**)—*E. indica*; (**E**)—*A. conyzoides*; (**F**)—*C. iria*; (**G**)—*E. hirta*, and (**H**)—*C. difformis*.

**Figure 9:**
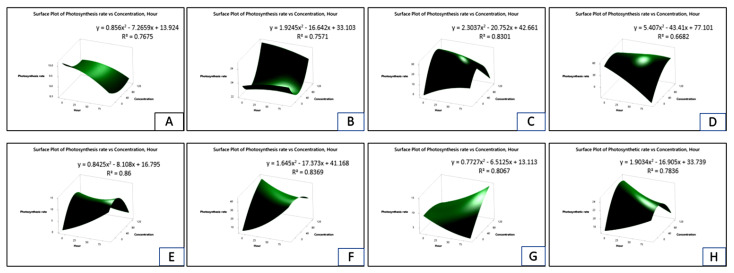
Response surface plot for photosynthesis rate of several crops and weeds due to parthenium leaf methanol extract: (**A**)—Bambara groundnut; (**B**)—Maize; (**C**)—*D. sanguinalis*; (**D**)—*E. indica*; (**E**)—*A. conyzoides*; (**F**)—*C. iria*; (**G**)—*E. hirta*, and (**H**)—*C. difformis*.

The rate of reduction was found dependent on the exposure time after the application of parthenium leaf extract. The extent of reduction in photosynthetic rates in the weed species was as follows: 75.18% in *D. sanguinalis*, 83.37% in *E. indica*, 58.58% in *A. conyzoides*, 51.71% in *C. iria*, 51.93% in *E. hirta*, and 22.60% in *C. difformis*, which was observed for the highest concentration (100 g L^−1^) at 6 HAS. The impacts of parthenium extract were more severe at 72 HAS, having the following sequence: *D. sanguinalis* (98.71%), *E. indica* (99.82%), *A. conyzoides* (70.27), *C. iria* (71.96%), *E. hirta* (75.35%) and *C. difformis* (26.11%), respectively (Figure 8 and Figure 9 and Tables S1–S5). It can be noted that in the case of crop species and the weed *C. difformis*, the decline in photosynthetic rate was comparatively less at 72 HAS.

### 2.8. Effect on Stomatal Conductance of Crops and Weeds

**Figure 10:**
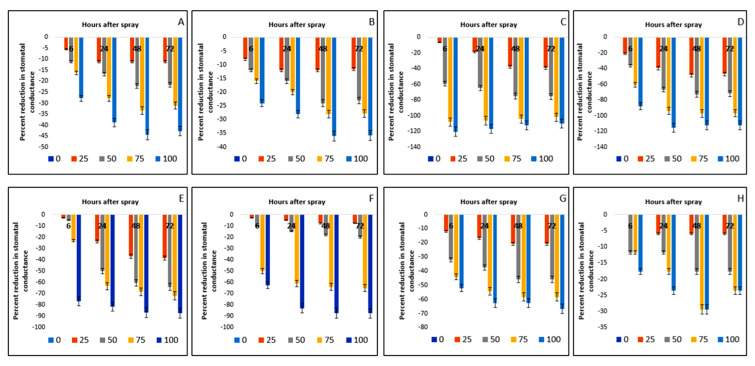
Reduction (%) of stomatal conductance of several crops and weeds due to the effect of parthenium leaf methanol extract: (**A**)—Bambara groundnut; (**B**)—Maize; (**C**)—*D. sanguinalis*; (**D**)—*E. indica*; (**E**)—*A. conyzoides*; (**F**)—*C. iria*; (**G**)—*E. hirta*, and (**H**)—*C. difformis*.

The stomatal conductance of the crops and the weeds were affected at different hours after spraying (Table 5). Among the test species, at a certain period after spraying, there was a noticeable difference in reaction to extract concentration levels. In Bambara groundnut, stomatal conductance was found to be lower (0.17 mol m^−2^ s^−1^) as in maize (0.25 mol m^−2^ s^−1^). On the other hand, comparatively higher values of stomatal conductance were noted in the weed species, especially *D. sanguinalis* (0.48 mol m^−2^ s^−1^), *E. indica* (0.32 mol m^−2^ s^−1^), *A. conyzoides* (0.38 mol m^−2^ s^−1^), and *C. iria* (0.39 mol m^−2^ s^−1^). The species *E. hirta* (0.24 mol m^−2^ s^−1^) and *C. difformis* (0.17 mol m^−2^ s^−1^) were weeds that were less affected. When a comparison is made between control plants and treated plants, the Bambara groundnut was found to be more affected than maize. The percent reductions in stomatal conductance in weed species due to application of parthenium extract at the rate of 100 g L^−1^ and measured at 6 HAS were 120.40%, 87.87%, 76.92%, 62.50%, 52%, and 17.64% in *D. sanguinalis*, *E. indica*, *A. conyzoides*, *C. iria*, *E. hirta*, and *C. difformis,* respectively (Figure 10). Again, *C. difformis* was least affected in its stomatal conductance. 

### 2.9. Effect on Transpiration Rate of the Crops and Weeds

**Figure 11:**
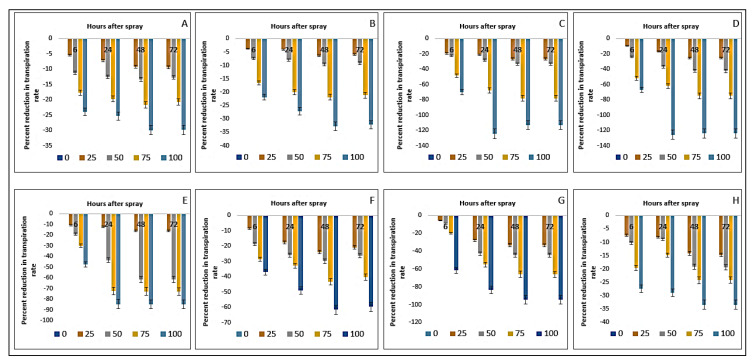
Reduction (%) of transpiration rate of several crops and weeds due to the eefect of parthenium leaf methanol extract: (**A**)—Bambara groundnut; (**B**)—Maize; (**C**)—*D. sanguinalis*; (**D**)—*E. indica*; (**E**)—*A. conyzoides*; (**F**)—*C. iria*; (**G**)—*E. hirta*, and (**H**)—*C. difformis*.

The transpiration rate of the crop and the weed species was also influenced negatively by the foliar spray of parthenium extract (Table 5). The crop species were comparatively less affected than the weed species. The rate of reduction in transpiration in the weeds was as follows: 124.58% in *D. sanguinalis,* 125.55% in *E. indica*, 84.77% in *A. conyzoides*, 61.60% in *C. iria*, 94.57% in *E. hirta,* and 33.37% in *C. difformis*, observed for the highest dose of extract and measured at 48 HAS (Figure 11). Therefore, the species *D. sanguinalis* and *E. indica* showed the highest inhibition in transpiration rate.

### 2.10. Effect on Malondialdehyde Content of the Crops and Weeds

The amount of malondialdehyde (MDA) in the control plants was much lower than in the *P. hysterophorus* extract-treated plants for all of the crops and weed species (Table 6). 

**Figure 12:**
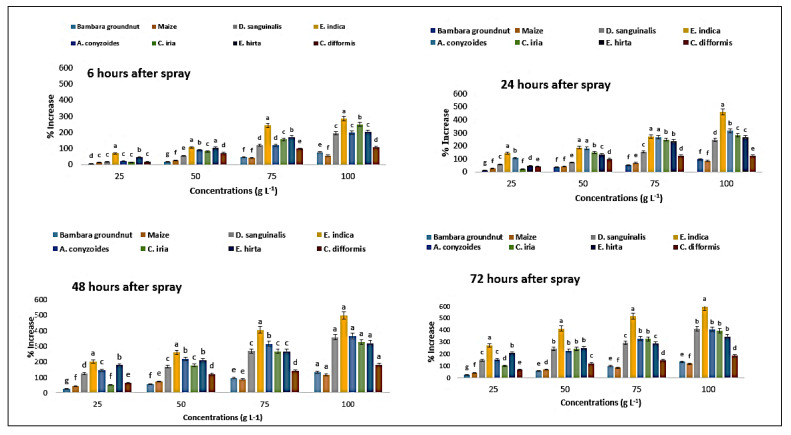
Increase (%) in MDA content in test crops and weeds treated with parthenium leaf methanol extract at different associated times. Values with the same letter among the tested crops and weeds are not significantly different at the same extract concentrations at *p* ≤ 0.005, determined by Turkey’s HSD.

In reaction to *P. hysterophorus* extract, MDA content was increased in a concentration-dependent manner. MDA content of Bambara groundnut was increased by 7.5 to 77.5% from the lowest concentrations (25 g L^−1^) to maximum (100 g L^−1^), respectively, at 6 HAS, and it was 14 to 57.14% for maize (Figure 12). After a shorter exposure (6 HAS) of *P. hysterophorus* extract to the lowest dose, the MDA level of test weeds remained unaltered. Longer exposure times (24, 48, and 72 HAS) resulted in significantly greater MDA levels than shorter exposure times (6 HAS) to *P. hysterophorus* extract (Figure 12). However, the MDA levels of *D. sanguinalis* were 409% higher than the control (0 g L^−1^) at 72 HAS, and 596% in *E. indica*, 404% in *A. conyzoides*, 391% in *C. iria*, 345% in *E. hirta*, and 183% in *C. difformis* at 72 HAS with higher concentration (100 g L^−1^). Among the weeds, *C. difformis* showed lower MDA content compared to other weeds, and *E. indica* showed higher MDA content. 

### 2.11. Effect on Proline Content of the Crops and Weeds

**Figure 13:**
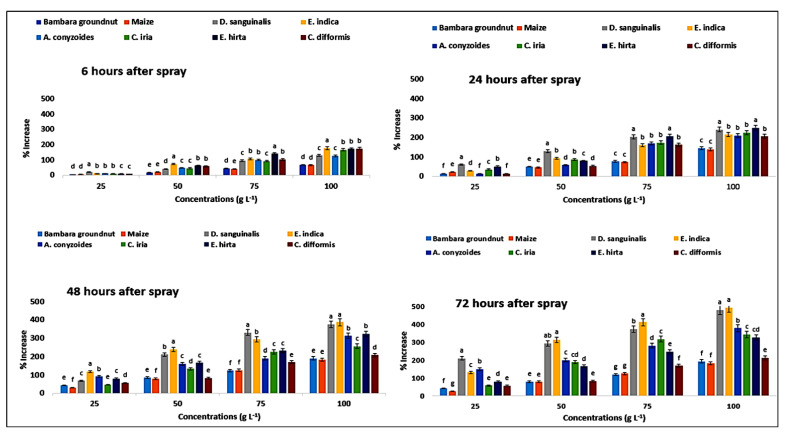
Increase (%) in proline content in test crops and weeds treated with leaf methanol extract of parthenium at the different associated times. Values with the same letter among the tested crops and weeds are not significantly different at the same extract concentrations at *p* ≤ 0.005, by Turkey’s HSD.

The content of proline was measured at six different exposure times (6, 24, 48, and 72 HAS) and found to differ considerably (Table 6). In reaction to *P. hysterophorus* extract, the content of proline increased in a concentration-dependent way. Proline content of Bambara groundnut was increased by 40 to 193% from the lowest (25 g L^−1^) to maximum (100 g L^−1^) concentrations, respectively, at 72 HAS, compared to control (0 g L^−1^), and 25 to 182% for maize (Figure 13). After a shorter exposure (6 HAS) to the lowest dose of *P. hysterophorus*, the proline content of the test weeds remained unaltered. Longer exposure times (24, 48, and 72 HAS) resulted in significantly greater MDA levels than shorter exposure times (6 HAS) in the case of *P. hysterophorus* extract (Figure 13). The response of crop and weed species to parthenium extract with respect to proline content was more or less similar to MDA content (Figure 13). Among the weeds, *E. indica* showed the highest proline content for 6, 24 and 48 HAS compared to other weeds at 72 HAS, next to *D. sanguinalis* with the highest concentration.

### 2.12. Effect on Superoxide Dismutase Content of the Crops and Weeds

**Figure 14:**
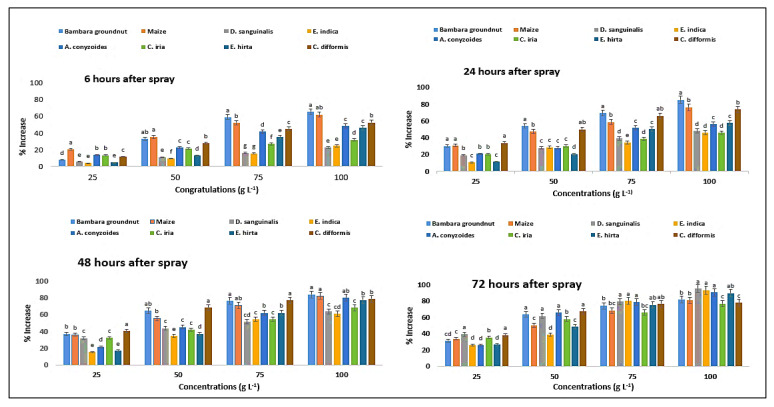
Increase (%) content of SOD in test crops and weeds treated with parthenium leaf methanol extract at different associated times. Values with the same letter among the tested crops and weeds are not significantly different at the same extract concentrations at *p* ≤ 0.005, by Tukey’s HSD.

The activity of superoxide dismutase (SOD) enzyme in the test plants, e.g., Bambara groundnut, maize, *D. sanguinalis*, *E. indica*, *A. conyzoides*, *C. iria*, *E. hirta*, and *C. difformis,* was significantly influenced by foliar spray of *P. hysterophorus* extract (Table 7). SOD activity was found to be higher with the application of *P. hysterophorus* leaf methanol extract (100 g L^−1^). In Bambara groundnut, the values were 65%, 85%, 84%, and 81.81%, whereas in maize these were 61, 76, 82, and 80% at 6, 24, 48, and 72 HAS, respectively, when the extract was applied at the rate of 100 g L^−1^ (Figure 14). On the other hand, the values were 22, 48, 63, and 95% in *D. sanguinalis*; 24, 46, 61, and 93% in *E. indica*; 48, 56, 80, and 90% in *A. conyzoides*; 31, 46, 68, and 76% in *C. iria*; 46, 57, 93, and 112% in *E. hirta*; and 52, 73, 78, and 78.05% in *C. difformis,* due to the application of parthenium extract at the rate of 100 g L^−1^ and measured at 6, 24, 48, and 72 HAS.

### 2.13. Effect on Catalase Content of the Crops and Weeds

**Figure 15:**
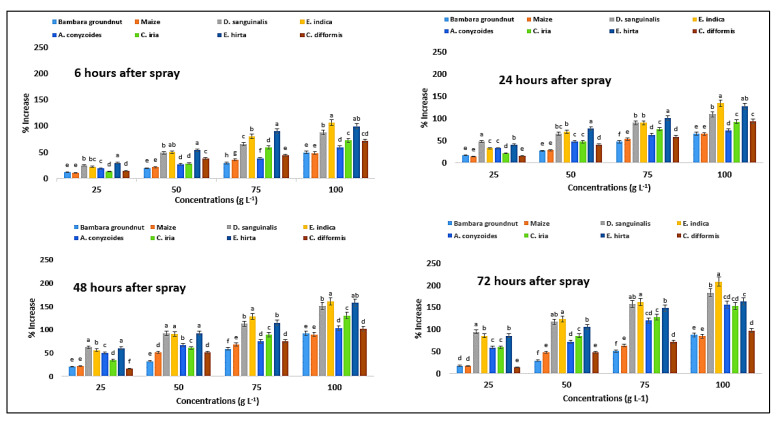
Percent increase in catalase (CAT) content of test crops and weeds treated with parthenium leaf methanol extract concentrations at different associated times. Values with the same letter among the tested crops and weeds are not significantly different at the same extract concentrations at *p* ≤ 0.005, by Tukey’s HSD.

At varied exposure durations, the activity of catalase (CAT) differed considerably with increasing concentration of *P. hysterophorus* extract (Table 7). The activity of CAT in Bambara groundnut, maize, *D. sanguinalis*, *E. indica, A. conyzoides, C. iria, E. hirta,* and *C. difformis* was considerably increased by foliar spray of *P. hysterophorus* extract at different times. In Bambara groundnut, the values were 49, 66, 91, and 87%, but in maize the values were 48, 65, 89, and 85%, measured at 6, 24, 48, and 72 HAS, respectively, with the concentration of 100 g L^−1^ (Figure 15). 

On the other hand, the values in the weed species were higher, such as 88, 110, 150, and 183% in *D. sanguinalis*; 106, 134, 160, and 208% in *E. indica*; 60, 72, 103, and 157% in *A. conyzoides*; 73, 93, 130, and 153% in *C. iria*; 100, 128, 157, and 163% in *E. hirta*; and 71, 94, 101, and 98% in *C. difformis,* when measured at 6, 24, 48, 72 HAS, respectively, with the concentration of 100 g L^−1^. The highest activity of CAT enzymes was observed at 72 HAS from *E. indica*, followed by *D. sanguinalis* and *E. hirta*, with the highest concentration of *P. hysterophorus* leaf methanol extract.

### 2.14. Effect on Peroxidase Content of the Crops and Weeds

**Figure 16:**
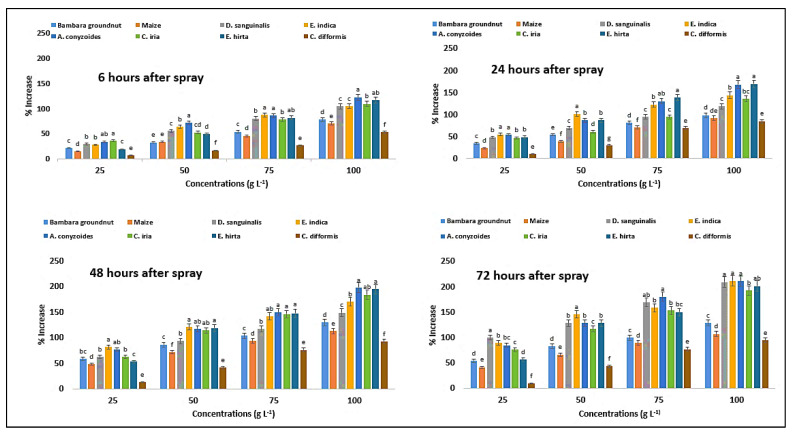
Increase (%) of peroxidase (POD) in several crops and weeds due to the foliar application of *pathenium* leaf methanol extract concentrations at the different associate times. Values with the same letter among the tested crops and parthenium weeds are not significantly different at the same extract concentrations at *p* ≤ 0.005 by Tukey’s HSD.

The peroxidase (POD) contents of both the crops and weeds were also significantly influenced by the interaction of associated time and parthenium leaf extract concentrations (Table 6). The activity of POD increased linearly with the exposure durations and concentration levels (Figure 16). In Bambara groundnut, the values were 78%, 98%, 130%, and 128%, and in maize, these were 78%, 92%, 112%, and 107% at 6, 24, 48 and 72 HAS, respectively, with the concentration of 100 g L^−1^ (Figure 16). Again, the POD contents increased more in weeds than in crops, having values of 209% in *D. sanguinalis*, 212% in *E. indica,* 211% in *A. conyzoides,* 192% in *C. iria*, 201% in *E. hirta,* and 94% in *C. difformis* at 72 HAS, respectively, with the highest concentration of parthenium extract (100 g L^−1^). 

## 3. Discussion

Parthenium, in general, is a hazardous, poisonous, allergenic, pernicious, and aggressive weed that is a principal hazard to cattle, crops, and humans. The phytochemical screening revealed a huge number of compounds in the *P. hysterophorus* extracts, some of which have previously been identified as poisons in several investigations [31]. Furthermore, various plant sections of *P. hysterophorus* contained a different number of compounds. The quantity of toxic compounds was more in the leaf than in the other plant parts; as a result, the leaves have a stronger inhibitory effect. The suppressive influence of extracts, according to Verdeguer et al. [33] is determined by the extract’s chemical composition together with the plant sections to which it is applied. These findings are consistent with those of Javaid and Anjum [34] and Verma et al. [35] who discovered that parthenin and other phenolic acids such as caffeic acid, vanillic acid, anisic acid, chlorogenic acid, and para hydroxybenzoic acid are the most responsible for plant growth inhibition.

In this work, we examined the effects of *P. hysterophorus* extract on physiological and biochemical alterations in Bambara groundnut, maize, *D. sanguinalis*, *E. indica, A. conyzoides, C. iria, E. hirta,* and *C. difformis*. Chlorophyll contents in leaves were changed throughout the extract exposure time, which is a good measure of plant resistance and ecological sustenance [36]. Since photosynthesis is the fundamental process by which the light energy is absorbed and converted into organic matter in the presence of green pigments of plants, the role of the plant pigment, chlorophylls (a and b) as an intermediary in the process is important [37]. We observed a significant reduction in the extract-treated plants when compared to the control groups. The allelopathic impact of parthenium is responsible for the decrease in chlorophyll content. The reduced chlorophyll and carotenoid concentrations are proportional to the concentration of parthenium extract, which agrees with Algandaby and Salama [38], who discovered that a high concentration of allelochemicals reduces chlorophyll content, limiting photosynthesis and plant development. An increase in the chlorophyllase enzyme is associated with reduced chlorophyll content in stressful conditions [39]. Carotenoids protect the plants against free radicals and photochemical damage by acting as antioxidants [40,41]. When exposed to *Portulaca oleracea* root extract, reduced chlorophyll b and carotenoids were 81.40 and 77.8%, respectively, and this might be due to degradation of existing chlorophyll or a decrease in chlorophyll production [42]. 

The foliar spray of leaf extract of parthenium reduced the stomatal conductance, photosynthetic rate, and transpiration in both the test crop and weed species. Reduced leaf photosynthesis was ascribed to a decrease in carboxylation efficiency, photosynthetic metabolites, chloroplast impairment, and increased enzyme activities in mulberry plants [43]. The ROS formation hampered the photosynthetic mechanism [44]. Stomatal control is a crucial characteristic that allows plants to regulate water loss and maintain gas exchange. This trait can be altered by a variety of conditions, including chemical stresses [45]. In the plants exposed to parthenium extract, the efficiency of utilizing water and carboxylation was likewise lowered.

Stomatal conductance is unquestionably linked to a decrease in transpiration rate. The extract of parthenium leaf was found to play a significant role in lowering the transpiration rate of test plants at various exposure durations. Overall, water usage and transpiration of lettuce seedlings were decreased linearly as phenolic acid concentrations increased [46]. In a solution containing cinnamic and benzoic acids, cucumber seedlings displayed decreased stomatal conductance and transpiration [47]. 

Increased levels of malondialdehyde (MDA) in plant tissue are a good sign of membrane lipid peroxidation, which leads the plants to oxidative stress [48]. MDA levels were higher in parthenium-sprayed plants than in the control (no extract) plants, with the highest concentration showing the most apparent increase. Furthermore, higher levels of free radicals can lead to increased membrane lipid peroxidation and, as a result, higher MDA levels. MDA levels were greater in bean plants subjected to 20 mM cadmium than in controls [49]. In the presence of a high amount of MDA, the glufosinate-induced antioxidant enzymes may not be able to completely eliminate ROS in a short amount of time.

Proline is necessary for safeguarding cells against the formation of reactive oxygen species (ROS) under stressful conditions, in addition to osmotic adjustments. In response to drought and salinity, plants build protective compatible solutes, such as proline, to help them to absorb more water [50]. To fight against diverse abiotic stresses, plants have evolved ways to store appropriate solutes such as betaine, sugar, polyol, and proline, with proline being the most essential solute that reduces the impact of osmotic adjustment [51]. The treatment with parthenium extract resulted in the highest concentration of proline at 72 HAS. The proline content was dependent on the concentration of parthenium leaf extract and the exposure time in all crops and weeds. A similar result was observed by [52], who found that the proline content of three wheat cultivars was dramatically increased after applying *Medicago sativa* leaf extracts to the leaves. The concentration of proline in wheat, tomato, and cucumber treated with *Calotropis procera* aqueous extract was also increased [53].

The first line of defensive antioxidants, which primarily include superoxide dismutase (SOD), catalase (CAT), and glutathione peroxidase (GPX), take a significant and indispensable part in the overall antioxidant defense strategy. Parthenium exposures increased the amounts of SOD, POD, and CAT in the test plant species. Superoxide dismutase (SODs) is the initial step in the elimination of reactive oxygen species (ROS). They catalyze the conversion of superoxide into oxygen and hydrogen peroxide. As a result, increases in SOD activity in response to parthenium extract trigger enhanced O_2_ generation. Similarly, higher POD and CAT activity suggest that these antioxidant enzymes may protect against oxidation [54]. The activities of SOD, POD, and CAT in the leaves of weeds, e.g., *D. sanguinalis, E. indica, A. conyzoides, C. iria,* and *E. hirta,* were significantly higher than *C. difformis* at higher concentrations. When plants were exposed to a stressful condition, the activity of one or more of these enzymes generally increased [55]. It could be because of the interruption of their infrastructure [56]. 

The enzymatic activity of selected weeds such as *D. sanguinalis, E. indica, A. conyzoides, C. iria,* and *E. hirta* were dramatically increased due to foliar spray of parthenium extract at the rate of 100 g L^−1^ and measured at 72 HAS. POD increased dramatically, indicating a greater level of H_2_O_2_ that is produced as a derivative of SOD metabolism. In general, the activity of enzymes rises as the concentration of parthenium extract rises. During the first time parthenium extract was used, it appeared that the enzymes (SOD, CAT, and POD) in the organelles, notably mitochondria and chloroplasts, were insufficient to scavenge ROS and prevent membrane oxidation. Furthermore, chlorotic symptoms produced in response to allelochemical stress indicate the activation signal molecule and production of defense molecules. 

## 4. Materials and Methods

### 4.1. Experimental Site

**Figure 17:**
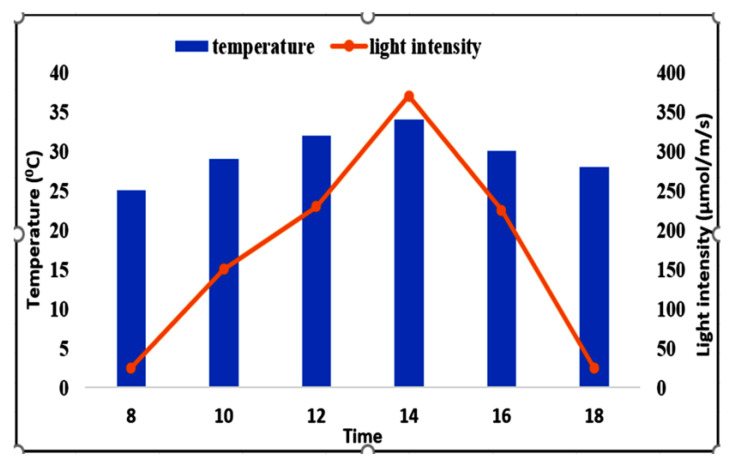
Temperature and light intensity fluctuation in the glasshouse. Note: The temperature was measured using a thermometer and light intensity was measured by a heavy-duty light meter (Extech^®^ model 407026).

A pot experiment was conducted from April to July 2021 in a glasshouse of the Faculty of Agriculture, Universiti Putra Malaysia (3°02′ N, 101°42′ E, 31 m elevation), and in the Weed Science Laboratory, UPM, Selangor, Malaysia. The local climate is hot, with high humidity and abundant rainfall. The average daily temperature and light intensity inside the glasshouse were recorded at hourly intervals (Figure 17). 

### 4.2. Experimental Treatments and Design

**Figure 18:**
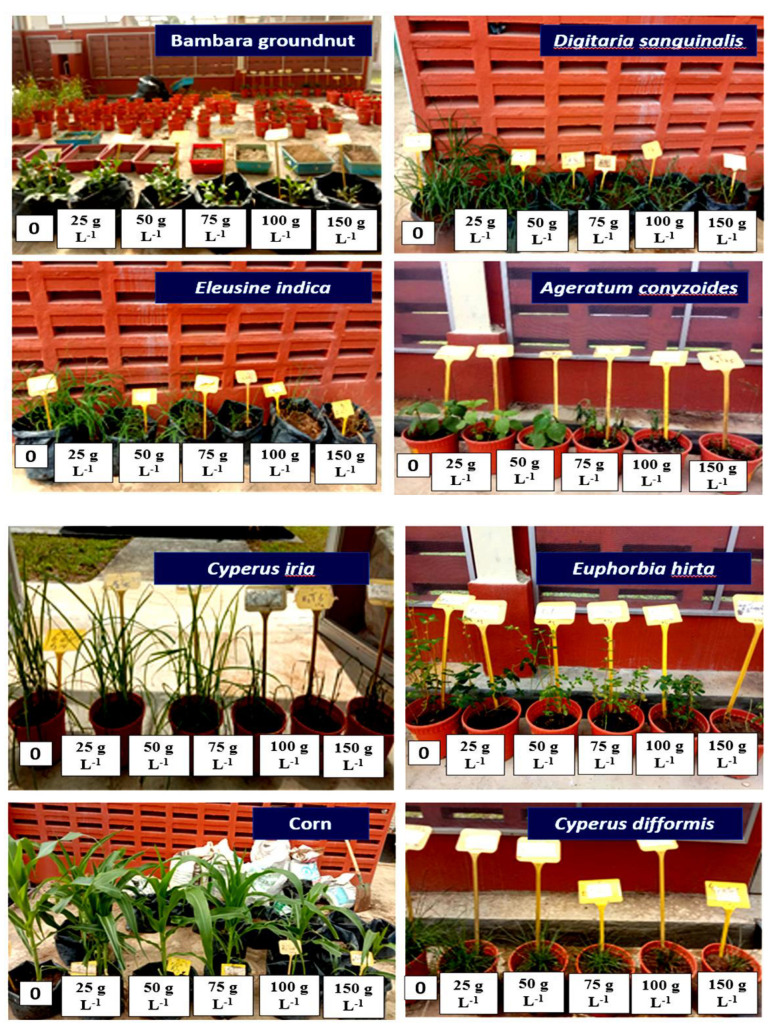
Effect of parthenium leaf methanol extract after post-emergence application on different crops and weeds.

The pots were organized in a four-replication randomized complete block design (RCBD). Treatments included leaf methanol extract of *P. hyterophorus* at the concentrations of 25, 50, 75, and 100 g L^−1^ and 0 (water, as control) (Figure 18).

### 4.3. Extract Preparation

Parthenium (leaves, stem, and flower) were collected from Ladang Infoternak farm in Sungai Siput, Perak, Malaysia, and grown in the nethouse of Field-15 at Universiti Putra Malaysia, Selangor, Malaysia. Before maturing, parthenium plants were collected and rinsed with tap water numerous times to remove dust particles, and air-dried for three weeks at room temperature (24–26 °C). The leaves, stems, and flowers were separated into three major components. In a laboratory blender, plant leaves were mashed into a fine powder and sieved through a 40-mesh sieve. 

The extracts were made according to the procedure described in [57]. An amount of 100 g powder of parthenium leaf, stem, and flower was placed in a conical flask and allowed to soak in 1 L of 80% (*v/v*) methanol. The conical flask was wrapped in paraffin and shaken for 48 h at 24–26 °C (room temperature) in an orbital shaker at 150 rpm agitation speed. To remove debris, cheesecloth in four layers was used to filter the mixtures. The supernatant was centrifuged for one hour at 3000 rpm in a centrifuge (5804/5804 R, Eppendorf, Germany). A single layer of Whatman No. 42 filter paper was used to filter the supernatant. A 0.2 mm Nalgene filter (Lincoln Park, NJ, Becton Dickinson percent Labware) was used to filter the solutions one more time to exclude microbial development. Using a rotary evaporator (R 124, Buchi Rotary Evaporator, Germany), the solvents were evaporated from the extract to dryness (a thick mass of coagulated liquid) under vacuum at 40 °C, and the sample was then collected. From a 100 g sample of *P. hysterophorus* powder, the average extracted sample was 17.56 g, which was estimated as per the following formula [58]:

Extract weight (g)/powder weight (g)] *×* 100 = Extraction percentage (1)

The stock of extract of parthenium leaves was diluted appropriately with sterile distilled water to generate extract concentrations of 25, 50, 75, and 100 g L^−1^ and water (distilled) was used as a control. All extracts were stored at 4 °C in the dark until use. 

For LC-MS analysis, 100% HPLC GRADE methanol (20 mL) was diluted with the crude sample (20 mg) and filtered through 15 mm, 0.2 µm syringe filters (Phenex, Non-sterile, Luer/Slip, and LT Resources Malaysia).

### 4.4. Identification of Phytochemicals in Different Parthenium Plant Parts Extracted in Methanol

LC-MS was used to identify the chemical contents of the extracts. Analysis of the phytochemical compounds in the methanol extracts was performed using LC-MS following the methods in [59]. LC-MS analysis was performed using an Agilent spectrometer equipped with a binary pump. The LC-MS was interfaced with the Agilent 1290 Infinity LC system coupled to an Agilent 6520 accurate-mass Q-TOF mass spectrometer with a dual ESI source. Full-scan mode from m/z 50 to 500 was performed with a source temperature of 125 °C. An Agilent zorbax eclipse XDB-C18 column, narrow-bore 2.1 × 150 mm, 3.5 microns (P/N: 930990-902), was used at the temperature 30 °C for the analysis. A: 0.1% formic acid in water, and B: 0.1% formic acid in methanol were used as solvents. Isocratic elution was used to supply solvents at a total flow rate of 0.1 mL minutes^−1^. MS spectra were collected in both positive and negative ion modes. The drying gas was 300 °C, with a 10 mL min^−1^ gas flow rate and a 45 psi nebulizing pressure. Before analysis, 1 mL of concentrated sample extract was diluted with methanol and filtered through a 0.22 m nylon filter. The extracts were injected into the analytical column in 1 µL volume for analysis. The mass fragmentations were discovered using an Agilent mass hunter qualitative analysis B.07.00 (Metabolom-ics-2019.m) tool and a spectrum database for organic chemicals.

### 4.5. Quantification of Phytotoxic Compounds through HPLC Analysis

#### 4.5.1. Chemicals

The chemicals Caffeic, Ferulic, Vanillic, Quinic acid, Parthenin, Chlorogenic and Anisic acid together with electronic-grade Methanol and Acetonitrile were acquired from Sigma-Aldrich (USA), while Merck (India) provided the HPLC grade water. Extracts were prepared from the aerial parts of *P. hysterophorus* L following the method used by Niranjan et al. [60]. 

#### 4.5.2. Preparation of Stock Solutions and Extracts

Primary stock solutions of methanolic extract (1 mg mL^−1^) were prepared, which were diluted with methanol to prepare solutions with different concentrations ranging from 0.5 to 50 mg mL^−1^. All of the solutions were kept in a refrigerator at 4 °C for further use.

#### 4.5.3. Analysis of Compounds

HPLC-Dionex (Germany) was used to do qualitative and quantitative analyses for chemical separation of extract using a Chromeleon system (California, USA) with a dual pump system. Compounds were separated using a C18 (Phenomenex) column (250 mm × 4.6 mm) with 5 mm pore size and a guard column with the same packing material [61]. A gradient of acetic acid (1%, *v/v*) in HPLC-grade water (component A) and acetonitrile was used as the mobile phase (component B). The components were kept free from air bubbles by using an ultrasonic bath before being filtered through 0.45 µm nylon filters. Elutions of component B were from 18 to 36% in 0–15 min, and from 36–50% in 15–40 min at a flow rate of 1.0 mLmin^−1^.

Data were integrated by Chromeleon 6.8 chromatography data system software and results were obtained by comparison with standards. All samples and solutions were filtered through 0.45 µm nylon filters (Millipore) before analysis by HPLC. MeOH mobile phase was used as the control for the identification of blank peaks. Before injecting into HPLC, 1 mg of residue was dissolved in 2 mL electronic-grade methanol. By comparing peak areas of samples with those of standards, the content of organic acids was determined in mgg^−1^ (dry weight). The mean values of three replicates of the same sample were calculated.

### 4.6. Test Plants and Methodology

The test crops were Bambara groundnut, maize, and weeds: *D. sanguinalis, E. indica, A. conyzoides, C. iria, E. hirta,* and *C. difformis*. The seeds of weeds were collected from Field 15, Universiti Putra Malaysia, and crop seeds were collected from Sin Seng Huat Seed SdnBhd, Selangor, Malaysia. The weed seeds were dried properly and kept in the laboratory at 4 ℃ for 15 days before use. Pre-germinated test plant seeds were placed in each plastic pot (18 cm diameter × 18 cm height), which were then topped with 5 cm of soil and saturated with tap water. Only five equal-sized healthy seedlings each of *D. sanguinalis, E. indica, A. conyzoides, C. iria, E. hirta,* and *C. diformis* and one equal-sized healthy seedling each of Bambara groundnut and maize were kept in each pot after germination. With the use of a 1 L multipurpose sprayer (Deluxe pressure sprayer), 100 mL m^−2^ leaf extracts of parthenium were sprayed on the weed seedlings at 2–3 leaf stage (2 weeks old) for grasses and 4–6 leaf stage (3 weeks old) for broadleaf species [62]. In this way, maintaining two pots in 1 m^2^ area, and 50 mL of leaf extract was required for each pot retaining five plants. In the control treatment only, distilled water was sprayed at the rate of 200 mL per pot at two-day intervals, or when needed.

### 4.7. Data Collections

The test plants’ photosynthetic rate, transpiration, and stomatal conductance, their chlorophyll fluorescence, chlorophyll pigments, proline, and antioxidant enzymes were documented after parthenium leaf extract was applied. These were measured to elucidate the potential mechanism of its allelopathy. Two to three leaf samples of individual test crops and weeds were taken at 6, 24, 48 and 72 hours of spraying. After that, these were wrapped in aluminum foil and put in an icebox, and transported to the laboratory from the glasshouse and promptly frozen using liquid nitrogen before being stored at −80 °C for biochemical analysis.

#### 4.7.1. Determination of Photosynthetic Rate, Stomatal Conductance, and Transpiration Rate

Between 9.00 and 11:00 a.m. in clear daylight 15 days after sowing, LICOR (LI-6400XT) portable photosynthesis equipment (LI-COR-Inc. Lincoln, Lincoln, NE, USA) was used to quantify photosynthesis, transpiration rate, and stomatal conductance from four randomly selected remaining leaves in each test crop and weed species. The experiments were carried out on the abaxial surface with a CO_2_ flow rate of 400 mol m^−2^ s^−1^ and a saturating photosynthetic photon flux density (PPFD) of 1000 mmol m^−2^ s^−1^ [63].

#### 4.7.2. Estimation of Chlorophyll Pigments

The carotenoids and total chlorophyll contents were assessed using the procedure described in [61,64]. Fresh leaf samples (0.1 g) were homogenized in 10 mL of 80% acetone in a glass bottle. The glass bottles were wrapped in aluminum foil and stored at room temperature for three days. Two milliliters of homogenized sample was transferred to several test tubes. The test tubes were vortexed when the incubation was completed and the sediments had settled to the bottom. The absorbance of the solution was measured using a spectrophotometer (UV-3101 P, Labomed Inc., Los Angeles, CA, USA) at the wavelengths of 663.2, 646.8, and 470 nm, and 80% acetone only was used as a blank. The following relationships were used to calculate the amounts of chlorophyll-a, chlorophyll-b, total chlorophyll, and carotenoids in mg g^−1^ of fresh weight (FW) [65,66,67,68]:

(2) $$\mathrm{Chlorophyll}-a\left(\mu {\mathrm{gmL}}^{-1}\right)=\left(12.25\times A663.2-2.79\times A646.8\right)$$

(3) $$\mathrm{Chlorophyll}-b\left(\mu {\mathrm{gmL}}^{-1}\right)=\left(21.50\times A646.8-5.1\times A663.2\right)$$

(4) $$\mathrm{Total}\;\mathrm{chlorophyll}\;\left(\mu {\mathrm{gmL}}^{-1}\right)=\left(7.15\times A663.2+18.71\times A646.8\right)$$

(5) $$\mathrm{Carotenoids}\;\left(\mu {\mathrm{gmL}}^{-1}\right)=\frac{\left(1000\times A470-1.8\times \mathrm{chl}a-85.02\times \mathrm{chl}b\right)}{198}$$

where A is absorbance.

#### 4.7.3. Measurement of Malondialdehyde (MDA) Content 

The malondialdehyde (MDA) content was measured using the methodology stated by [69]. Two milliliters (2 mL) of ultrapure distilled water was used to homogenize 0.2 g of crushed leaf tissue. The materials were then centrifuged at 10,000 rpm for 15 min (Sigma 3K30). One milliliter of this solution was heated in a water bath at 90 °C for 30 min with two milliliters of thiobarbituric acid (TBA) + trichloroacetic acid (TCA) (Merck, Germany) solution (0.5% TBA in 20% TCA). After boiling, the test tubes containing the solution were chilled in an ice bath. The resulting mixture was centrifuged at 10,000 rpm for 15 min. The spectrophotometric absorbance of the supernatants (UV-3101PC, Shimadzu) was measured at 450, 532, and 600 nm. The concentration of MDA was calculated using the formula below [9]:

MDA (µM) = {6.45 *×* (D532 − D600) − 0.56 *×* D450} (6)

The absorbance at 450, 532, and 600 nm, respectively, were D450, D532, and D600. Finally, the MDA content was calculated as mol g^−1^ FW.

#### 4.7.4. Measurement of Proline Content

The proline quantity was determined by using the procedures reported in Bates et al. [70] with minor modifications. In the presence of 2 mL of 5% (*w/v*) sulfosalicylic acid, 0.1 g of fresh leaves was homogenized. Sample-containing examination tubes were centrifuged at 10,000 rpm for 10 min; 1 mL of the supernatant was combined with 1 mL of acid ninhydrin (30 mL glacial acetic acid; 1.25 g of ninhydrin; 20 mL phosphoric acid 6 M) and 1 mL of glacial acetic acid. After that, the solution-filled tubes were placed in a water bath for an hour at 95 °C before cooling for 10 min in an ice bath. Each tube was filled with two milliliters of toluene, and the samples were agitated with a vortex. Using a microplate reader, an absorbance reading at 520 nm was taken to determine the proline concentration (Bio Tek 800 TS). Proline content was determined by the value in a standard curve with L-proline as the standard (Sigma-Aldrich, St. Louis, MO, USA), and then using the equation below:

(7) $$\mathrm{Proline}\;\left(\mu {\mathrm{molg}}^{-1}\mathrm{FW}\right)=\frac{\frac{\;\mathrm{Proline}\;\left(\mu {\mathrm{gmL}}^{-1}\right)\times \;\mathrm{Tolune}\;\left(\mathrm{mL}\right)}{115.5\left(\mu g\mu {\;\mathrm{mole}\;}^{-1}\right)}}{\;\mathrm{Freshweightofsample}\;\left(g\right)}$$

where, the proline molecular weight is 115.5 (µg µmole*^−^*^1^).

#### 4.7.5. Estimation of Enzymes 

The antioxidant enzymes, superoxide dismutase (SOD), catalase (CAT), and peroxidase (POD), were estimated using the procedure described in [71,72] to assess their activity. Liquid nitrogen was used to grind a fresh leaf sample in a porcelain mortar. Then, 1.5 mL potassium phosphate buffer was added to a 0.1 g powdered leaf sample in a 2 mL Eppendorf tube. The mixture was centrifuged at 10,000 rpm for 20 min. The following parameters were then measured with the prepared samples.

#### 4.7.6. Determination of SOD

The sample was made according to the instructions in [71,72]. To make the sample, 0.05 mL enzyme extract, 0.1 mL 3 mM EDTA, 1.5 mL potassium phosphate buffer, 0.1 mL 200 mM methionine, 1 mL ultrapure distilled water, and 0.01 mL 2.25 mM NBT (n-nitro blue tetrazolium) were mixed properly. Finally, in the dark, 60 M riboflavin was added to each reaction mixture in test tubes. The test tubes were then incubated for 10 min under a 15 watt fluorescent bulb. The blanks comprised reaction mixes that included no enzyme extract and were not kept under the light after incubation to halt the reaction. The tubes were covered with aluminum foil, and SOD was measured at 560 nm using a spectrophotometer (UV-3101PC). The changes in absorbance of enzyme extracts caused by the reaction of the superoxide nitro blue tetrazolium complex were recorded. The amount of enzyme that inhibits NBT reduction to 50% was used to determine each unit of enzyme activity using the following formula:

(8) $$\mathrm{SOD}\;(\%\;\mathrm{inhibition})=\frac{\left(A560\;\mathrm{control}\;-\;A560\;\mathrm{sample}\right)\times 100}{\;A560\;\mathrm{control}\;}$$

(9) $$\mathrm{SOD}(\mathrm{Unit}\left.{\mathrm{mL}}^{-1}\right)=\frac{\%\;\mathrm{inhibition}\;\times \;\mathrm{total}\;\mathrm{volume}\;}{50\times \;\mathrm{enzyme}\;\mathrm{volume}\;}$$

(10) $$\mathrm{SOD}(\mathrm{Unit}\left.{\mathrm{mg}}^{-1}\mathrm{FW}\right)=\frac{{\;\mathrm{unit}\;\mathrm{mL}}^{-1}}{\;\mathrm{enzyme}\;\left({\mathrm{mgmL}}^{-1}\right)}$$

The absorbances of sample and control were recorded at the wavelength of 560 nm for 1 min, and one unit of SOD generation is equal to 50% inhibition. The activity of SOD was expressed as unit mg*^−^*^1^ FW.

#### 4.7.7. Determination of CAT

The CAT activity was investigated according to the Khan et al. [73] and Aebi [74] approaches. The reaction mixture was prepared to a volume of 3.0 mL by adding 1.5 mL of 100 mM potassium phosphate buffer, 0.05 mL of enzyme extract, 0.5 mL of 75 mM hydrogen peroxide (H_2_O_2_), and 0.95 mL of ultrapure distilled water. The mixture that did not contain any enzyme extract was referred to as “blank”. The blank solution was placed in a spectrophotometer for 4 to 5 min to achieve temperature equilibration. The absorbance was measured for 2 min at a wavelength of 240 nm in a spectrophotometer (UV-3101PC, Shimadzu, Japan). The quantity of catalase enzyme activity that decomposes 1 M H_2_O_2_ was calculated for each unit of catalase enzyme activity. The catalase activity was measured in milligrams per gram of fresh tissue (Min^−1^ g^−1^ FW).

(11) $$\mathrm{CAT}\;\left(\mu {\mathrm{molmin}}^{-1}{\mathrm{mL}}^{-1}\right)=\frac{\left(A240/\min\right)\times \;\mathrm{total}\;\mathrm{volume}\;\times 1000}{43.6\times \;\mathrm{enzyme}\;\mathrm{volume}\;}$$

(12) $$\begin{array}{c} \;\mathrm{CAT}\;\left(\mu {\mathrm{molmin}}^{-1}{\mathrm{mg}}^{-1}\mathrm{FW}\right)=\frac{\mu {\mathrm{molmin}}^{-1}{\mathrm{mL}}^{-1}}{\;\mathrm{enzyme}\;\left({\mathrm{mgmL}}^{-1}\right)} \end{array}$$

The sample’s absorbance was measured at 240 nm for 1 min, and the extinction coefficient was 43.6.

#### 4.7.8. Determination of POD

The POD action was calculated using the techniques of [75], and was assessed by H_2_O_2_ with the oxidation of guaiacol at 470 nm. An amount of 0.1 mL enzyme extract, 2.83 mL 10 mM phosphate buffer (pH 7.0), and 0.05 mL 20 mM guaiacol were combined in a 3 mL reaction mixture, while the reaction was started with 0.02 mL of 40 mM H_2_O_2_. The mixture that did not contain any enzyme extract was referred to as “blank”. The blank solution was placed for 4 to 5 min to achieve temperature equilibration in a spectrophotometer. The absorbance was determined at a wavelength of 470 nm, and the POD unit was determined as mol min^−1^ g^−1^ FW.

(13) $$\mathrm{POD}\;\left(\mu {\mathrm{molmin}}^{-1}{\mathrm{mL}}^{-1}\right)=\frac{\left(A470/\min\right)\times \;\mathrm{total}\;\mathrm{volume}\;\times 1000}{26.6\times \;\mathrm{enzyme}\;\mathrm{volume}\;}$$

(14) $$\mathrm{POD}\left(\mu {\mathrm{molmin}}^{-1}{\mathrm{mg}}^{-1}\mathrm{FW}\right)=\frac{\mu {\mathrm{molmin}}^{-1}{\mathrm{mL}}^{-1}}{\;\mathrm{enzyme}\;\left({\mathrm{mgmL}}^{-1}\right)}$$

The sample’s absorbance was measured at 470 nm for 1 min, and the extinction coefficient was 26.6.

### 4.8. Statistical Analysis

To determine whether there was a statistically significant difference between each treatment and control, a two-way analysis of variance (ANOVA) was carried out using R-studio software. The mean comparison was computed using least significant difference (LSD) at a significance level of *p* ≤ 0.05. Response surface regression analysis was performed by using Minitab statistical software. The percent reduction and percent increasing were calculated by comparison to the control.

## 5. Conclusions

The results from this study suggested that the crude extracts contained a sufficient amount of phytochemicals/allelochemicals, which were responsible for their medicinal and toxicological properties. Seven phytochemicals such as caffeic acid, ferulic acid, vanillic acid, parthenin, chlorogenic acid, quinic acid, and p-anisic acid were identified from the *P. hysterophorus* leaf extract, which was responsible for inhibition. All the identified compounds were detected at higher amounts in leaf methanol extracts (40,752.52 ppm) than those of the stem (2664.09 ppm) and flower extracts (30,454.33 ppm). Parthenium leaf methanol extracts were found to be responsible for alterations in the physiological and biochemical parameter in crops such as Bambara groundnut and maize, and in weed species, e.g., *D. sanguinalis, E. indica, A. conyzoides, C. iria, E. hirta,* and *C. difformis.* Although both the crops and the weeds were affected by the foliar sprays of parthenium extract at 100 g L^−1^, the symptoms of stressed conditions were recovered easily after a few days in crops but not in the weeds. The extract amounts elicited diverse reactions in the species, which could be related to the species’ innate genetic heterogeneity. The application of extract resulted in a decrease in chlorophyll content and carotenoids, which, as a result, reduced the photosynthesis rate. The activity of antioxidant enzymes (e.g., SOD, CAT, and POD) and MDA and proline content, depend on parthenium extract. This condition presents an opportunity to control the weeds that have developed resistance to current herbicides. The information from the study and advanced methods of chemistry and biochemistry, together with new molecular genetics, proteomics, and metabolomics profiling tools, will be helpful in developing a new bioherbicide for selective and ecofriendly control of weeds, based on the structure of potential natural herbicidal compounds from parthenium. 

## Figures and Tables

**Table 1:** Phenolic derivatives found in methanol extract of different parts of *Parthenium hysterophorus* through LC-MS analysis.

Sl No.	Compound Name	Synonyms	Chemical Formula	Retention Time	m/z	Mass	Polarity	Chemical Structure	Biological Activity	Plant Part			References
										Leaf	Stem	Flower	
1.	Caffeic acid	3-4-Dihydroxy cinnamic acid	C_9_H_8_O_4_	7.183	341.0894	342.09698	Negative	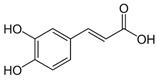	Antifungal, dermatitis, autotoxic, inhibitory effect to other plants	+	-	+	[29,30,31,32]
		3-(3,4-dihydroxy phenyl) acrylic acid											
2.	Ferulic acid	Trans-ferulic acid	C_10_H_10_O_4_	9.84	193.05129	194.05855	Negative	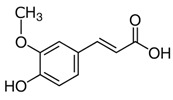		+	-	+	
		4-hydroxy-3-methoxy cinnamic acid											
		Coniferic acid											
		2 Propenoic acid, 3-(4-hydroxy-3-methoxy phenyl)											
3.	Vanillic acid	4-hydroxy-3-methoxy benzoic acid	C_8_H_8_O_4_	7.367	153.01983	154.02711	Negative	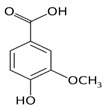		+	+	+	
		Benzoic acid, 4-hydroxy-3-methoxy											
4.	Quinic acid	D-(-)-Quinic acid	C_7_H_12_O_6_	12.116	181.12	180.1129	Positive	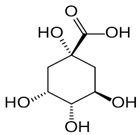		+	-	+	
		Chinic acid											
		Quinate											
		1,3,4,5-tetrahydroxy cyclohexane carboxylic acid											
5.	Parthenin	10-alpha-H-Ambrosia-2,11(13)-1,6-beta di-hydroxy-4-oxo-,gamma –lactone	C_15_H_18_O_4_	10.004	263.1267	262.1194	Positive	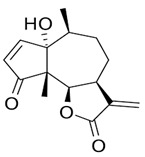		+	+	+	
		Grosshemin											
		Helenalin											
6.	Chlorogenic acid	3,0-caffeoylquinic acid	C_16_H_18_O_9_	8.09	300.183	282.1421	Positive	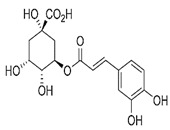		+	-	+	
		3-(3,4-dihydroxy cinnamoyl) quinic acid											
		3-caffeoylquinic acid											
		1,3,4,5-tetrahydroxy cyclohexane carboxylic acid											
7.	*p*-Anisic acid	4-methoxy benzoic acid	C_8_H_8_O_3_	5.121	151.04047	152.04764	Negative	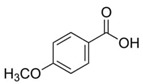		+	-	+	
		p-anisic acid											
		p-methoxy benzoic acid											

Note: “+” symbol indicates present; “-“symbol indicates absent; m/z indicates mass number/charge number means mass-to-change ratio.

**Table 2:** Major phytotoxic compounds and their quantity (ppm) detected from aerial parts extracts of *P. hysterophorus*.

No.	Retention Time (min)	Detected Compounds	Leaf Methanol Extract	Stem Methanol Extract	Flower Methanol Extract
			Amount (ppm)		
1.	4.19	Parthenin	4208.08	2650.76	3823.67
2.	5.64	Quinic acid	36,504.48	-	26,528.56
3.	6.89	Chlorogenic acid	17.841	-	65.270
4.	7.20	Vanillic acid	5.149	13.334	2.431
5.	7.35	Caffeic acid	7.635	-	0.278
6.	9.00	Ferulic acid	7.807	-	28.519
7.	9.62	Anisic acid	1.535	-	5.609
Total amount (ppm)			40,752.52	2664.09	30,454.33

**Table 3:** Response of chlorophyll-*ɑ* and chlorophyll-*ɓ* content due to the foliar spray of *P. hysterophorus* leaf extract on several crops and weeds.

Test Plants	Concentration (g L^−1^)	Chlorophyll-*ɑ* (mg g^−1^ FW)				Chlorophyll-*ɓ* (mg g^−1^ FW)			
		Hours after Spray				Hours after Spray			
		6	24	48	72	6	24	48	72
Bambara groundnut	0	3.24aA ± 0.08	3.16aA ± 0.02	3.04aB ± 0.03	2.93aC ± 0.01	3.91aA ± 0.02	3.83aB ± 0.01	3.80aB ± 0.01	3.90aA ± 0.01
	25	3.24aA ± 0.02	3.06bB ± 0.02	2.98aC ± 0.01	2.83bD ± 0.01	3.90aA ± 0.08	3.72bB ± 0.01	3.74bB ± 0.01	3.88aA ± 0.01
	50	2.96bA ± 0.07	2.92cA ± 0.01	2.79bB ± 0.01	2.78cB ± 0.01	3.40bA ± 0.03	3.29cB ± 0.01	3.26cC ± 0.02	3.53cB ± 0.07
	75	2.74cB ± 0.06	2.85dA ± 0.01	2.61cC ± 0.01	2.57dD ± 0.01	3.30cB ± 0.04	3.13dC ± 0.03	3.05dD ± 0.02	3.43cA ± 0.01
	100	2.64cB ± 0.01	2.70eA ± 0.01	2.27dC ± 0.01	2.19eC ± 0.01	3.22dB ± 0.01	2.99eC ± 0.01	2.94eD ± 0.01	3.28dA ± 0.01
Maize	0	3.11aA ± 0.01	2.62aB ± 0.01	2.46aD ± 0.02	2.58aC ± 0.01	4.94aA ± 0.02	4.84aB ± 0.03	4.94aA ± 0.02	4.88aB ± 0.03
	25	2.90bA ± 0.03	2.57bB ± 0.01	2.37aC ± 0.01	2.55bB ± 0.01	4.85bA ± 0.04	4.65bC ± 0.02	4.65bC ± 0.06	4.72bB ± 0.03
	50	2.89bA ± 0.06	2.42cB ± 0.02	2.30aC ± 0.02	2.42cB ± 0.01	4.77cA ± 0.02	4.47cC ± 0.01	4.44cC ± 0.05	4.51cB ± 0.01
	75	2.84bA ± 0.05	2.33dC ± 0.02	2.23aC ± 0.69	2.35dB ± 0.01	4.75cA ± 0.01	4.34dB ± 0.01	4.13dC ± 0.34	4.34dB ± 0.02
	100	2.54cA ± 0.07	2.28eB ± 0.01	2.12bC ± 0.01	2.27eB ± 0.01	4.44dA ± 0.01	4.14eC ± 0.04	4.01eD ± 0.05	4.28eB ± 0.02
*D. sanguinalis*	0	3.18aA ± 0.01	3.04aB ± 0.02	2.54aC ± 0.03	2.53aC ± 0.01	5.61aC ± 0.02	5.62aC ± 0.01	5.69aB ± 0.04	5.91aA ± 0.02
	25	2.88bA ± 0.02	2.78bB ± 0.01	2.43bC ± 0.01	2.38bD ± 0.01	5.33bA ± 0.02	5.09bB ± 0.05	5.10bB ± 0.06	4.66bC ± 0.01
	50	2.68cA ± 0.01	2.65cA ± 0.02	1.81cB ± 0.01	1.68cC ± 0.02	4.90cA ± 0.03	3.93cB ± 0.01	3.97cB ± 0.03	3.49cC ± 0.06
	75	2.39dA ± 0.01	2.36dA ± 0.01	1.28dB ± 0.02	1.24dC ± 0.01	3.35dA ± 0.05	2.92dB ± 0.03	2.61dC ± 0.01	2.00dD ± 0.02
	100	2.01eA ± 0.01	1.43eB ± 0.02	0.99eC ± 0.02	0.51eD ± 0.01	2.44eA ± 0.02	1.65eB ± 0.01	1.49eC ± 0.04	1.09eD ± 0.02
*E. indica*	0	3.10aA ± 0.02	3.04aB ± 0.01	2.81aC ± 0.03	2.79aC ± 0.02	5.70aB ± 0.03	5.59aC ± 0.01	5.57aC ± 0.01	5.87aA ± 0.03
	25	3.00bA ± 0.01	2.49bB ± 0.01	2.32bC ± 0.01	1.93bD ± 0.01	4.61bA ± 0.02	4.46bB ± 0.06	4.23bC ± 0.01	3.67bD ± 0.01
	50	2.68cA ± 0.02	2.43cB ± 0.01	2.03cC ± 0.01	1.04cD ± 0.01	4.36cA ± 0.02	3.73cB ± 0.00	3.56cC ± 0.02	3.38cD ± 0.03
	75	2.54dA ± 0.01	1.97dB ± 0.01	1.21dC ± 0.01	0.85dD ± 0.01	3.89dA ± 0.03	3.61dB ± 0.01	2.87dC ± 0.01	2.32dD ± 0.03
	100	2.07eA ± 0.01	1.29eB ± 0.02	0.94eC ± 0.01	0.59eD ± 0.01	2.79eA ± 0.02	2.69eB ± 0.04	1.55eC ± 0.02	1.12eD ± 0.02
*Ageratum conyzoides*	0	3.01aA ± 0.01	2.96aB ± 0.01	2.83aC ± 0.003	2.85aC ± 0.01	0.96aB ± 0.06	0.89aC ± 0.05	0.86aC ± 0.04	1.00aA ± 0.03
	25	2.70bA ± 0.03	1.58bB ± 0.02	1.51bC ± 0.02	1.25bD ± 0.01	0.91aA ± 0.01	0.72bB ± 0.03	0.71bB ± 0.03	0.70bB ± 0.02
	50	2.02cA ± 0.03	1.44cB ± 0.03	1.27cC ± 0.01	1.09cD ± 0.06	0.74bA ± 0.01	0.70bA ± 0.10	0.67bB ± 0.03	0.61cC ± 0.03
	75	1.79dA ± 0.01	1.32dB ± 0.004	1.12dC ± 0.08	0.97dD ± 0.01	0.55cA ± 0.05	0.55cA ± 0.05	0.49cAB ± 0.05	0.45cB ± 0.02
	100	1.71eA ± 0.02	1.22eB ± 0.01	1.03eC ± 0.06	0.82eD ± 0.01	0.49cA ± 0.03)	0.45cA ± 0.02	0.39dB ± 0.02	0.26dC ± 0.01
*C. iria*	0	3.28aA ± 0.02	3.07aB ± 0.06	2.53aD ± 0.02	3.00aC ± 0.01	3.67aC ± 0.01	3.66aC ± 0.02	3.91aA ± 0.02	3.79aB ± 0.03
	25	3.22bA ± 0.01	3.05aB ± 0.01	2.52aD ± 0.01	2.66bC ± 0.01	2.62bA ± 0.04	2.33bB ± 0.02	2.10bC ± 0.05	1.82bD ± 0.02
	50	3.18cA ± 0.01	2.97bB ± 0.02	2.41bC ± 0.01	2.28cD ± 0.01	1.89cA ± 0.04	1.81cB ± 0.03	1.85cAB ± 0.03	1.55cC ± 0.01
	75	3.01dA ± 0.01	2.83cB ± 0.03	2.13cC ± 0.03	1.04dD ± 0.02	1.74dA ± 0.02	1.53dB ± 0.01	1.49dC ± 0.01	1.42dD ± 0.02
	100	2.47eA ± 0.01	2.20dB ± 0.04	1.04dC ± 0.04	0.84eD ± 0.01	1.74dA ± 0.02	1.37eB ± 0.02	1.31eC ± 0.03	1.20eD ± 0.02
*E. hitra*	0	1.23aB ± 0.01	1.08aC ± 0.01	0.96aD ± 0.02	1.32aA ± 0.01	2.27aAB ± 0.04	2.32aA ± 0.02	2.34aA ± 0.03	2.23aB ± 0.03
	25	−0.37bA ± 0.01	−0.34bA ± 0.01	−0.29bB ± 0.01	−0.10bC ± 0.01	−0.52bA ± 0.02	−0.45bB ± 0.02	−0.43bB ± 0.01	−0.36bC ± 0.01
	50	−0.38bA ± 0.00	−0.38cA ± 0.01	−0.31bB ± 0.01	−0.21cC ± 0.02	−0.68cA ± 0.07	−0.67cA ± 0.07	−0.60cAB ± 0.02	−0.52cB ± 0.01
	75	−0.41cA ± 0.02	−0.43dA ± 0.01	−0.44cA ± 0.02	−0.27dB ± 0.01	−0.84dA ± 0.01	−0.70cB ± 0.02	−0.62cC ± 0.01	−0.55cD ± 0.01
	100	−0.64dA ± 0.01	−0.61eB ± 0.01	−0.58dC ± 0.01	−0.33eD ± 0.01	−0.90dA ± 0.04	−0.82dB ± 0.03	−0.70dC ± 0.02	−0.69dC ± 0.02
*C. difformis*	0	3.36aA ± 0.02	3.14aB ± 0.01	2.71aD ± 0.01	3.04aC ± 0.01	2.86aA ± 0.04	2.73aB ± 0.03	2.86aA ± 0.03	2.86aA ± 0.03
	25	3.08bA ± 0.01	2.95bC ± 0.03	2.66bD ± 0.01	3.00bB ± 0.01	2.85bA ± 0.04	2.65bC ± 0.01	2.81bB ± 0.03	2.82bB ± 0.02
	50	3.01cA ± 0.02	2.96bB ± 0.03	2.63cC ± 0.01	2.96cB ± 0.02	2.75cA ± 0.02	2.49cC ± 0.03	2.60cB ± 0.02	2.61cB ± 0.03
	75	3.00cA ± 0.01	2.77cC ± 0.02	2.52dD ± 0.01	2.82dB ± 0.01	2.61dA ± 0.03	2.38dC ± 0.01	2.42dB ± 0.05	2.43dB ± 0.03
	100	2.89dA ± 0.04	2.47dC ± 0.03	1.96eD ± 0.03	2.51eB ± 0.10	2.48eA ± 0.01	2.25eB ± 0.03	2.12eD ± 0.01	2.20dC ± 0.03

Data are expressed as means ± standard error. Mean with the same small letters in the column for each concentration and the capital letter within the hours are not significantly different at *p* ≤ 0.05.

**Table 4:** Response of total chlorophyll content and carotenoids as a consequence of foliar spray of *P. hysterophorus* leaf extract on several crops and weeds.

Test Plants	Concentration (g L^−1^)	Total Chlorophyll (mg g^−1^ FW)				Carotenoids (mg g^−1^ FW)			
		Hours after Spray				Hours after Spray			
		6	24	48	72	6	24	48	72
Bambara groundnut	0	7.15aA ± 0.09	6.99aB ± 0.04	6.84aC ± 0.05	6.83aC ± 0.02	1.56aA ± 0.003	1.55aB ± 0.00	1.40aC ± 0.00	1.55aB ± 0.001
	25	7.14bA ± 0.06	6.78bB ± 0.01	6.72bC ± 0.02	6.71bC ± 0.01	1.54bA ± 0.003	1.50bB ± 0.00	1.33bD ± 0.002	1.47bC ± 0.001
	50	6.36cA ± 0.05	6.21cC ± 0.02	6.05cD ± 0.03	6.31cB ± 0.09	1.45cA ± 0.001	1.38cB ± 0.001	1.23cD ± 0.003	1.35cC ± 0.001
	75	6.04dA ± 0.10	5.98dB ± 0.02	5.66dC ± 0.02	6.00dA ± 0.004	1.39dA ± 0.002	1.32dB ± 0.002	1.15dD ± 0.002	1.28dC ± 0.001
	100	5.86eA ± 0.01	5.69eB ± 0.03	5.21eD ± 0.07	5.47eC ± 0.02	1.321eA ± 0.001	1.23eB ± 0.001	1.11eC ± 0.001	1.23eB ± 0.003
Maize	0	8.05aA ± 0.03	7.46aB ± 0.04	7.40aC ± 0.04	7.46aB ± 0.04	1.69aA ± 0.001	1.67aB ± 0.001	1.63aC ± 0.001	1.69aA ± 0.001
	25	7.75bA ± 0.07	7.22bC ± 0.03	7.02bD ± 0.07	7.27bB ± 0.04	1.64bA ± 0.001	1.61bB ± 0.001	1.54bC ± 0.002	1.64bA ± 0.001
	50	7.66cA ± 0.08	6.89cC ± B0.02	6.74cD ± 0.07	6.93cB ± 0.02	1.60cA ± 0.001	1.55cB ± 0.00	1.44cC ± 0.002	1.55cB ± 0.00
	75	7.59dA ± 0.05	6.67dC ± 0.04	6.36dD ± 0.35	6.69dB ± 0.03	1.52dA ± 0.001	1.42dC ± 0.00	1.34dD ± 0.01	1.46dB ± 0.001
	100	6.98eA ± 0.06	6.42eC ± 0.05	6.13eD ± 0.07	6.55eB ± 0.03	1.46eA ± 0.001	1.34eC ± 0.002	1.29eD ± 0.002	1.38eB ± 0.001
*D. sanguinalis*	0	8.80aA ± 0.03	8.67aB ± 0.03	8.23aD ± 0.07	8.45aC ± 0.03	1.73aA ± 0.001	1.70aD ± 0.001	1.71aC ± 0.001	1.72aB ± 0.001
	25	8.21bA ± 0.04	7.87bB ± 0.06	7.53bC ± 0.07	7.04bD ± 0.02	1.45bA ± 0.001	1.40bB ± 0.002	1.35bC ± 0.002	1.33bD ± 0.001
	50	7.58cA ± 0.04	6.58cB ± 0.04	5.65cC ± 0.05	5.30cD ± 0.06	1.23cA ± 0.001	1.17cB ± 0.001	1.01cC ± 0.001	0.94cD ± 0.002
	75	5.71dA ± 0.07	5.32dB ± 0.04	3.90dC ± 0.03	3.25dD ± 0.03	0.98dA ± 0.002	0.85dB ± 0.001	0.50dC ± 0.00	0.47dD ± 0.001
	100	4.45eA ± 0.03	3.08eB ± 0.02	2.48eC ± 0.06	1.60eD ± 0.03	0.80eA ± 0.001	0.53eB ± 0.00	0.39eC ± 0.001	0.11eD ± 0.001
*E. indica*	0	8.80aA ± 0.05	8.63aB ± 0.02	8.38aC ± 0.05	8.66aB ± 0.05	1.27aA ± 0.001	1.25aB ± 0.00	1.22a ± 0.00	1.26aB ± 0.001
	25	7.47bA ± 0.06	7.11bB ± 0.04(17.61)	6.56bC ± 0.02	5.60bD ± 0.02	1.26bA ± 0.002	1.18bB ± 0.001	1.18bB ± 0.00	1.02bC ± 0.00
	50	7.05cA ± 0.04	6.17cB ± 0.02(28.50)	5.41cC ± 0.04	4.60cD ± 0.03	1.10cA ± 0.001	1.03cB ± 0.00	0.93cC ± 0.001	0.81cD ± 0.001
	75	6.43dA ± 0.05	5.59dB ± 0.02	4.08dC ± 0.02	3.18dD ± 0.04	0.88dA ± 0.001	0.78dB ± 0.00	0.60dC ± 0.00	0.47dD ± 0.001
	100	4.76eA ± 0.06	4.09eB ± 0.05	2.50eC ± 0.03	1.71eD ± 0.03(80.25)	0.83eA ± 0.002	0.51eB ± 0.001	0.47eC ± 0.001	0.31eD ± 0.001
*Ageratum conyzoides*	0	3.88aA ± 0.05	3.88aA ± 0.06	3.73aB ± 0.06	3.86aA ± 0.05	1.17A ± 0.001	1.16aB ± 0.002	1.16aB ± 0.002	1.17aA ± 0.001
	25	3.31bA ± 0.06	2.55bB ± 0.03	2.22bC ± 0.04	1.93bD ± 0.05	1.06A ± 0.001	0.87bB ± 0.001	0.80bC ± 0.001	0.75bD ± 0.001
	50	2.73cA ± 0.07	2.18cB ± 0.03	1.98cC ± 0.12	1.82cD ± 0.09	0.91A ± 0.001	0.72cB ± 0.00	0.65cC ± 0.004	0.61cD ± 0.001
	75	2.35dA ± 0.06	1.86dB ± 0.05	1.62dC ± 0.12	1.43dD ± 0.03	0.76dA ± 0.002	0.72cB ± 0.002	0.64dC ± 0.002	0.55dD ± 0.001
	100	2.11eA ± 0.04	1.71eB ± 0.04	1.49dC ± 0.08	1.09eD ± 0.02	0.69eA ± 0.001	0.54dB ± 0.001	0.42eC ± 0.001	0.35eD ± 0.001
*C. iria*	0	6.95aA ± 0.03	6.73aB ± 0.09	6.44aC ± 0.04	6.79aB ± 0.04	1.60aA ± 0.00	1.58aB ± 0.001	1.54aC ± 0.001	1.60aA ± 0.001
	25	5.84bA ± 0.06	5.39bB ± 0.03	4.62bC ± 0.06	4.49bD ± 0.03	1.55bA ± 0.002	1.30bB ± 0.001	1.16bC ± 0.002	1.02bD ± 0.001
	50	5.07cA ± 0.06	4.78cB ± 0.05	4.26cC ± 0.04	3.83cD ± 0.02	1.22cA ± 0.001	1.17cB ± 0.001	1.09cC ± 0.001	0.96cD ± 0.001
	75	4.75dA ± 0.03	4.20dB ± 0.05	3.45dC ± 0.06	2.54dD ± 0.03	1.01dA ± 0.001	0.93dB ± 0.001	0.67dC ± 0.001	0.49dD ± 0.00
	100	4.21eA ± 0.03	3.74eB ± 0.06	2.47eC ± 0.07	2.05eD ± 0.03	0.92eA ± 0.001	0.71eB ± 0.001	0.48eC ± 0.001	0.43eD ± 0.001
*E. hitra*	0	3.51aA ± 0.06	3.41aB ± 0.03	3.30aC ± 0.05	3.56aA ± 0.04	0.79aB ± 0.001	0.78aC ± 0.001	0.75aD ± 0.001	0.80aA ± 0.001
	25	−0.81bA ± 0.04	−0.80dA ± 0.02	−0.79bA ± 0.03	−0.46bB ± 0.02	−0.09bA ± 0.001	−0.12bB ± 0.001	−0.05bC ± 0.001	−0.03eD ± 0.00
	50	−1.07cA ± 0.07	−1.05cA ± 0.07	−0.92cB ± 0.03	−0.73cC ± 0.03	−0.11cA ± 0.003	−0.11cB ± 0.001	−0.06cC ± 0.001	−0.05dD ± 0.00
	75	−1.26dA ± 0.03	−1.14cB ± 0.04	−1.07dC ± 0.03	−0.83dD ± 0.02	−0.13dA ± 0.001	−0.08dB ± 0.003	−0.08dC ± 0.001	−0.08cD ± 0.001
	100	−1.54eA ± 0.05	−1.44bB ± 0.04	−1.28eC ± 0.03	−1.03eD ± 0.03	−0.14eA ± 0.002	−0.07eB ± 0.001	−0.11eC ± 0.001	−0.08bD ± 0.001
*C. difformis*	0	6.22aA ± 0.06	5.87aB ± 0.05	5.57aC ± 0.04	5.91aB ± 0.04	1.64aA ± 0.001	1.58aD ± 0.001	1.61aC ± 0.001	1.63aB ± 0.001
	25	5.93bA ± 0.03	5.60bC ± 0.04	5.47bD ± 0.04	5.82bB ± 0.03	1.59bA ± 0.001	1.47bD ± 0.001	1.49bC ± 0.001	1.58bB ± 0.001
	50	5.76cA ± 0.04	5.45cC ± 0.06	5.23cD ± 0.03	5.57cB ± 0.05	1.52cA ± 0.001	1.38cD ± 0.001	1.40cC ± 0.001	1.45cB ± 0.001
	75	5.61dA ± 0.05	5.15dC ± 0.03	4.94dD ± 0.05	5.25dB ± 0.04	1.43dA ± 0.001	1.27dD ± 0.001	1.29dC ± 0.002	1.37dB ± 0.001
	100	5.37eA ± 0.04	4.72eB ± 0.06	4.08eD ± 0.03	4.71eC ± 0.05	1.38eA ± 0.00	1.18eC ± 0.001	1.13eD ± 0.001	1.25eB ± 0.001

Data are stated as means ± SE. Mean with the same small letters in the column for each concentration and the capital letter within the hours are not significantly different at *p* ≤ 0.05.

**Table 5:** Response of photosynthesis rate, stomatal conductance, and transpiration rate due to the foliar spray of *P. hysterophorus* leaf extracts on several crops and weeds.

Test Plants	Conc. (g L^−1^)	Photosynthesis Rate (µmol m^−2^ s^−1^)				Stomatal Conductance (mol m^−2^ s^−1^)				Transpiration Rate (mmol m^−2^ s^−1^)			
		Hours after Spray				Hours after Spray				Hours after Spray			
		6	24	48	72	6	24	48	72	6	24	48	72
Bambara groundnut	0	10.54aA ± 0.01	10.04aC ± 0.13	10.04aC ± 0.01	10.35aB ± 0.05	0.18aA ± 0.01	0.18aA ± 0.01	0.17aB ± 0.01	0.18aA ± 0.01	15.64aB ± 0.04	15.63aC ± 0.04	15.65aA ± 0.01	15.65aA ± 0.01
	25	10.34bA ± 0.01	9.82bB ± 0.06	9.36bD ± 0.06	9.78bC ± 0.01	0.17bA ± 0.01	0.16bB ± 0.01	0.16bB ± 0.01	0.16bB ± 0.01	14.79bA ± 0.02	14.51bB ± 0.02	14.20bC ± 0.01	14.19bD ± 0.01
	50	9.96cA ± 0.11	8.63cC ± 0.11	8.47cD ± 0.10	8.84cB ± 0.03	0.16cA ± 0.01	0.15cB ± 0.01	0.14cC ± 0.01	0.14cC ± 0.01	13.89cA ± 0.02	13.67cB ± 0.01	13.56cD ± 0.01	13.66cC ± 0.11
	75	9.29dA ± 0.04	8.27dB ± 0.13	7.52dD ± 0.01	8.09dC ± 0.02	0.15dA ± 0.01	0.13dB ± 0.01	0.12dC ± 0.01	0.12dC ± 0.00	12.87dA ± 0.01	12.55dB ± 0.01	12.26dD ± 0.01	12.42dC ± 0.01
	100	8.64eA ± 0.05	7.07eC ± 0.07	7.01eD ± 0.07	7.58eB ± 0.03	0.13eA ± 0.01	0.11eB ± 0.01	0.10eC ± 0.01	0.10eC ± 0.01	11.90eA ± 0.01	11.67eB ± 0.01	10.97eD ± 0.01	10.98eC ± 0.01
Maize	0	25.79aA ± 0.05	25.64aB ± 0.10	25.54aD ± 0.03	25.61aC ± 0.01	0.25aB ± 0.00	0.25aB ± 0.01	0.25aB ± 0.01	0.26aA ± 0.00	15.81aA ± 0.01	15.19aB ± 0.01	15.19aB ± 0.01	15.19aB ± 0.01
	25	25.69bA ± 0.02	24.40bB ± 0.13	23.86bD ± 0.02	24.32bC ± 0.03	0.23bA ± 0.01	0.22bB ± 0.00	0.22bB ± 0.01	0.23bA ± 0.01	15.21bA ± 0.01	14.57bB ± 0.01	14.23bD ± 0.01	14.28bC ± 0.01
	50	24.82cA ± 0.05	22.78cB ± 0.08	22.13cD ± 0.03	22.42cC ± 0.01	0.22cA ± 0.01	0.21cB ± 0.01	0.19cD ± 0.01	0.20cC ± 0.00	14.64cA ± 0.02	13.98cB ± 0.01	13.73cD ± 0.02	13.79cC ± 0.01
	75	23.12dA ± 0.02	19.46dB ± 0.16	19.12dD ± 0.02	19.37dC ± 0.02	0.21dA ± 0.00	0.20dB ± 0.01	0.18dD ± 0.01	0.19dC ± 0.01	13.19dA ± 0.01	12.14dB ± 0.01	11.87dD ± 0.01	11.98dC ± 0.01
	100	22.01eA ± 0.06	18.23eC ± 0.18	17.57eD ± 0.12	18.93eB ± 0.05	0.19eA ± 0.01	0.18eB ± 0.01	0.16eC ± 0.01	0.16eC ± 0.01	12.35eA ± 0.01	11.06eB ± 0.01	10.23eC ± 0.01	10.30eC ± 0.01
*D. sanguinalis*	0	38.97aA ± 0.07	33.26aB ± 0.13	33.17aB ± 0.05	32.74aC ± 0.04	0.49aA ± 0.01	0.48aAB ± 0.01	0.48aAB ± 0.01	0.49aA ± 0.01	15.19aA ± 0.01	15.17aB ± 0.01	15.16aBC ± 0.01	15.15aC ± 0.01
	25	32.85bA ± 0.04	28.25bB ± 0.02	25.82bC ± 0.04	23.50bD ± 0.05	0.46bA ± 0.01	0.39bB ± 0.01	0.30bC ± 0.01	0.30bC ± 0.01	12.21bA ± 0.01	11.95bB ± 0.02	11.12bC ± 0.01	11.10bC ± 0.01
	50	27.23cA ± 0.21	22.44cB ± 0.10	20.32cC ± 0.01	16.87cD ± 0.01	0.20cA ± 0.01	0.17cB ± 0.01	0.12cC ± 0.01	0.12cC ± 0.01	11.73cA ± 0.01	10.87cB ± 0.01	10.11cC ± 0.01	10.10cC ± 0.01
	75	19.04dA ± 0.21	15.11dB ± 0.12	8.86dC ± 0.03	4.57dD ± 0.01	−0.04dA ± 0.0	−0.03dB ± 0.00	−0.02dC ± 0.01	−0.01dD ± 0.0	7.84dA ± 0.01	4.84dB ± 0.01	3.34dC ± 0.02	3.33dC ± 0.01
	100	9.67eA ± 0.11	5.41eB ± 0.06	1.53eC ± 0.03	0.42eD ± 0.01	−0.10eA ± 0.0	−0.08eB ± 0.00	−0.06eC ± 0.01	−0.05eC ± 0.0	4.57eA ± 0.01	−3.73eB ± 0.01	−2.02eC ± 0.01	−2.02eC ± 0.01
*E. indica*	0	73.34aA ± 0.20	60.21aB ± 0.08	58.61aC ± 0.05	58.57aC ± 0.03	0.33aA ± 0.01	0.33aA ± 0.00	0.33aA ± 0.01	0.32aB ± 0.01	13.07aA ± 0.01	13.07aA ± 0.01	13.07aA ± 0.01	13.06aA ± 0.01
	25	59.25bA ± 0.23	51.18bB ± 0.06	42.41bC ± 0.03	38.87bD ± 0.03	0.26bA ± 0.01	0.20bB ± 0.01	0.17bC ± 0.01	0.17bC ± 0.01	11.80bA ± 0.01	10.86bB ± 0.01	9.77bC ± 0.01	9.76bC ± 0.01
	50	46.16cA ± 0.26	43.46cB ± 0.20	36.51cC ± 0.02	20.46cD ± 0.02	0.21cA ± 0.01	0.11cB ± 0.01	0.09cC ± 0.01	0.09cC ± 0.01	9.92cA ± 0.01	8.23cB ± 0.01	7.56cC ± 0.01	7.54cC ± 0.01
	75	27.40dA ± 0.05	22.56dB ± 0.11	7.50dC ± 0.05	4.38dD ± 0.01	0.13dA ± 0.01	0.02dB ± 0.01	0.01dC ± 0.01	0.01dC ± 0.01	6.32dA ± 0.01	4.97dB ± 0.01	3.30dC ± 0.01	3.29dC ± 0.01
	100	12.19eA ± 0.45	4.70eB ± 0.16	0.88eC ± 0.03	0.10eD ± 0.01	0.04eA ± 0.01	−0.05eB ± 0.01	−0.04eC ± 0.00	−0.04eC ± 0.0	4.27eA ± 0.01	−3.34eB ± 0.01	−3.12eC ± 0.01	−3.11eC ± 0.01
*geratum conyzoides*	0	15.60aA ± 0.26	13.36aB ± 0.53	10.12aC ± 0.01	10.16aC ± 0.02	0.39aA ± 0.01	0.38aB ± 0.01	0.38aB ± 0.01	0.39aA ± 0.01	8.10aA ± 0.01	8.09aB ± 0.01	8.08aBC ± 0.01	8.07aC ± 0.01
	25	13.89bA ± 0.09	10.24bB ± 0.10	9.82bC ± 0.03	8.76bD ± 0.02	0.38bA ± 0.01	0.29bB ± 0.01	0.24bC ± 0.01	0.24bC ± 0.01	7.21bA ± 0.01	7.09bB ± 0.01	6.77bC ± 0.01	6.77bC ± 0.01
	50	10.87cA ± 0.09	8.28cB ± 0.14	6.67cC ± 0.01	6.69cC ± 0.01	0.37bA ± 0.01	0.19cB ± 0.01	0.15cC ± 0.01	0.14cC ± 0.01	6.55cA ± 0.01	4.57cB ± 0.01	3.11cC ± 0.01	3.10cD ± 0.01
	75	8.53dA ± 0.19	6.11dB ± 0.12	4.13dC ± 0.05	3.95dC ± 0.03	0.30cA ± 0.01	0.14dB ± 0.01	0.12dC ± 0.01	0.11dC ± 0.01	5.67dA ± 0.01	2.23dB ± 0.01	2.21dBC ± 0.01	2.19dC ± 0.01
	100	6.46eA ± 0.27	3.27eBC ± 0.05	3.35eB ± 0.02	3.02eC ± 0.01	0.09dA ± 0.01	0.07eB ± 0.01	0.05eC ± 0.01	0.05eC ± 0.01	4.24eA ± 0.01	1.25eB ± 0.01	1.23eC ± 0.01	1.23eC ± 0.01
*C. iria*	0	41.44aA ± 0.28	38.88aB ± 0.11	37.51aC ± 0.02	37.38aC ± 0.03	0.40aAB ± 0.01	0.41aA ± 0.01	0.39aB ± 0.01	0.40aAB ± 0.00	14.09aA ± 0.01	13.07aB ± 0.01	12.97aC ± 0.01	12.34aD ± 0.01
	25	39.06bA ± 0.49	34.50bB ± 0.05	31.47bC ± 0.02	30.93bD ± 0.05	0.39aA ± 0.01	0.39bA ± 0.01	0.36b ± B0.01	0.37bB ± 0.00	12.88bA ± 0.01	10.77bB ± 0.01	9.86bC ± 0.01	9.76bD ± 0.01
	50	32.62cA ± 0.07	29.43cB ± 0.06	26.32cC ± 0.02	25.85cD ± 0.17	0.36bA ± 0.01	0.35cB ± 0.01	0.32cC ± 0.01	0.32cC ± 0.01	11.44cA ± 0.01	9.66cB ± 0.01	9.11cC ± 0.01	9.08cD ± 0.01
	75	24.45dA ± 0.13	20.99dB ± 0.11	18.77dC ± 0.02	16.89dD ± 0.01	0.20cA ± 0.01	0.16dB ± 0.01	0.14dC ± 0.01	0.14dC ± 0.00	10.06dA ± 0.01	8.77dB ± 0.01	7.35dC ± 0.01	7.36dC ± 0.01
	100	20.01eA ± 0.47	15.19eB ± 0.04	10.50eC ± 0.05	10.48eC ± 0.07	0.15dA ± 0.01	0.07eB ± 0.01	0.05eC ± 0.01	0.05eC ± 0.00	8.88eA ± 0.01	6.66eB ± 0.01	4.98eC ± 0.01	4.97eC ± 0.01
*E. hitra*	0	13.73aA ± 0.16	12.51aB ± 0.06	12.08aC ± 0.03	12.09aC ± 0.02	0.25aA ± 0.01	0.24aB ± 0.01	0.24aB ± 0.01	0.24aB ± 0.00	7.21aA ± 0.01	7.19aB ± 0.01	7.19aB ± 0.01	7.18aB ± 0.01
	25	11.55bA ± 0.11	9.53bB ± 0.05	8.31bC ± 0.01	8.32bC ± 0.02	0.22bA ± 0.01	0.20bB ± 0.01	0.19bB ± 0.01	0.19bB ± 0.01	6.80bA ± 0.01	5.18bB ± 0.01	4.80bC ± 0.01	4.77bD ± 0.01
	50	9.38cA ± 0.16	7.44cB ± 0.05	5.58cC ± 0.02	5.41cD ± 0.02	0.17cA ± 0.01	0.15cB ± 0.01	0.13cC ± 0.01	0.13cC ± 0.01	6.55cA ± 0.01	4.11cB ± 0.01	3.97cC ± 0.01	3.97cC ± 0.01
	75	6.60dA ± 0.16	5.71dB ± 0.04	4.25dC ± 0.02	4.10dC ± 0.01	0.14dA ± 0.01	0.11dB ± 0.01	0.10dC ± 0.01	0.10dC ± 0.00	5.76dA ± 0.01	3.22dB ± 0.01	2.45dC ± 0.01	2.43dD ± 0.01
	100	5.28eA ± 0.05	3.32eB ± 0.04	3.09eC ± 0.01	2.98eD ± 0.03	0.12eA ± 0.01	0.09eB ± 0.01	0.09dB ± 0.00	0.08eC ± 0.01	2.77eA ± 0.01	1.19eB ± 0.01	0.39eC ± 0.53	0.39eC ± 0.52
*C. difformis*	0	24.07aD ± 0.16	24.53aA ± 0.15	24.34aC ± 0.06	24.43aB ± 0.03	0.17aA ± 0.01	0.17aA ± 0.01	0.17aA ± 0.00	0.17aA ± 0.01	14.99aA ± 0.01	14.99aA ± 0.01	14.98aAB ± 0.01	14.97aB ± 0.01
	25	23.78bA ± 0.11	23.51bB ± 0.16	22.79bD ± 0.01	23.12bC ± 0.05	0.17aA ± 0.01	0.16bB ± 0.01	0.16bB ± 0.01	0.16bB ± 0.01	13.88bA ± 0.01	13.77bB ± 0.01	12.86bC ± 0.01	12.76bD ± 0.01
	50	22.49cB ± 0.22 (6.56)	22.47cC ± 0.11	21.31cD ± 0.02	21.51cA ± 0.05	0.15bA ± 0.01	0.15cA ± 0.01	0.14cB ± 0.01	0.14cB ± 0.00	13.44cB ± 0.01	13.66cA ± 0.01	12.11cC ± 0.01	12.08cD ± 0.01
	75	20.29dA ± 0.16	20.11dB ± 0.05	19.45dD ± 0.03	19.63dC ± 0.01	0.15bA ± 0.01	0.14dB ± 0.01	0.12dD ± 0.01	0.13dC ± 0.01	12.06dB ± 0.01	12.77dA ± 0.01	11.35dC ± 0.01	11.36dC ± 0.01
	100	18.63eA ± 0.34	18.54eB ± 0.04	17.53eD ± 0.02	18.05eC ± 0.06	0.14cA ± 0.01	0.13eB ± 0.01	0.12dC ± 0.01	0.13dB ± 0.01	10.88eA ± 0.01	10.66eB ± 0.01	9.98eC ± 0.01	9.98eC ± 0.00

Data are expressed as means ± standard error. Mean with the same small letters in the column for each concentration and the capital letter within the hours are not significantly different at *p* < 0.05.

**Table 6:** Response of malondialdehyde and proline content of some crops and weeds due to the foliar spray of *P. hysterophorus* leaf extract.

Test Plants	Concentration (g L^−1^)	Malondialdehyde Content (µmol g^−1^ FW)				Proline Content (µmol g^−1^ FW)			
		Hours after Spray				Hours after Spray			
		6	24	48	72	6	24	48	72
Bambara groundnut	0	0.40eB ± 0.02	0.41eA ± 0.01	0.38eB ± 0.02	0.38eB ± 0.01	1.78eA ± 0.07	1.76eC ± 0.02	1.77eB ± 0.04	1.74eD ± 0.04
	25	0.43dD ± 0.01	0.47dC ± 0.01	0.48dB ± 0.01	0.48dA ± 0.005	1.89dD ± 0.09	2.01dC ± 0.07	2.52dA ± 0.09	2.45dB ± 0.07
	50	0.48cC ± 0.01	0.57cB ± 0.01	0.59cB ± 0.01	0.59cA ± 0.02	2.12cD ± 0.07	2.65cC ± 0.09	3.28cA ± 0.07	3.11cB ± 0.07
	75	0.59bD ± 0.01	0.64bC ± 0.01	0.74bB ± 0.01	0.75bA ± 0.01	2.60bD ± 0.04	3.15bC ± 0.09	3.95bA ± 0.12	3.81bB ± 0.07
	100	0.71aD ± 0.01	0.81aC ± 0.01	0.88aB ± 0.01	0.89aA ± 0.01	3.01aD ± 0.09	4.34aC ± 0.09	5.14aA ± 0.11	5.11aB ± 0.09
Maize	0	0.07eA ± 0.01	0.07eA ± 0.01	0.07eA ± 0.01	0.06eA ± 0.01	1.26bA ± 0.02	1.24eB ± 0.04	1.22eC ± 0.02	1.24eB ± 0.07
	25	0.08dD ± 0.01	0.09dC ± 0.01	0.10dB ± 0.01	0.09dA ± 0.01	1.35abC ± 0.07	1.53dC ± 0.07	1.58dA ± 0.04	1.55dB ± 0.04
	50	0.09cD ± 0.01	0.10cC ± 0.01	0.12cB ± 0.01	0.10cA ± 0.01	1.56aD ± 2.68	1.82cC ± 0.07	2.18cB ± 0.04	2.21cA ± 0.07
	75	0.10bD ± 0.01	0.12bC ± 0.01	0.13bB ± 0.01	0.11bA ± 0.01	1.78abD ± 0.07	2.16bC ± 0.09	2.74bB ± 0.07	2.79bA ± 0.09
	100	0.11aD ± 0.01	0.13aC ± 0.01	0.15aB ± 0.01	0.13aA ± 0.01	2.11abD ± 0.07	2.98aC ± 0.07	3.45aB ± 0.07	3.50aA ± 0.10
*D. sanguinalis*	0	0.39eB ± 0.01	0.41dA ± 0.01	0.34eC ± 0.01	0.33eC ± 0.01	2.74eA ± 0.09	2.55eB ± 0.07	2.54eC ± 0.07	2.51eD ± 0.07
	25	0.46dD ± 0.02	0.66cC ± 0.01	0.76dB ± 0.01	0.82dA ± 0.01	3.35dD ± 0.07	4.14dC ± 0.07	4.25dB ± 0.07	7.79dA ± 0.09
	50	0.61cD ± 0.02	0.71cC ± 0.01	0.91cB ± 0.01	1.13cA ± 0.02	3.88cD ± 0.04	5.88cC ± 0.02	7.89cB ± 0.07	9.88cA ± 0.04
	75	0.86bC ± 0.01	1.05bB ± 0.05	1.25bA ± 0.01	1.29bA ± 0.12	5.37bD ± 0.07	7.75bC ± 0.04	10.91bB ± 0.07	11.86bA ± 0.04
	100	1.15aD ± 0.01	1.42aC ± 0.02	1.55aB ± 0.01	1.68aA ± 0.02	6.31aD ± 0.07	8.71aC ± 0.07	12.06aB ± 0.07	14.53aA ± 0.09
*E. indica*	0	0.27eA ± 0.01	0.27eA ± 0.01	0.27eA ± 0.01	0.25eB ± 0.01	2.00eD ± 0.02	2.18eA ± 0.07	2.14eB ± 0.04	2.11eC ± 0.57
	25	0.46dD ± 0.02	0.66dC ± 0.01	0.81dB ± 0.01	0.93dA ± 0.01	2.26dD ± 0.04	2.85dC ± 0.04	4.65dB ± 0.07	4.88dA ± 0.09
	50	0.56cD ± 0.01	0.78cC ± 0.02	0.97cB ± 0.01	1.28cA ± 0.01	3.48cD ± 0.07	4.23cC ± 0.07	7.23cB ± 0.02	8.72cA ± 0.04
	75	0.93bD ± 0.01	1.01bC ± 0.03	1.36bB ± 0.02	1.54bA ± 0.01	4.15bD ± 0.04	5.71bC ± 0.07	8.40bB ± 0.09	10.80bA ± 0.04
	100	1.04aD ± 0.01	1.51aC ± 0.01	1.61aB ± 0.01	1.74aA ± 0.01	5.57aD ± 0.07	6.92aC ± 0.07	10.42aB ± 0.07	12.51aA ± 0.07
*Ageratum conyzoides*	0	0.23eA ± 0.01	0.23eA ± 0.01	0.22eB ± 0.01	0.22eC ± 0.01	4.28eD ± 0.11	4.55eC ± 0.02	4.60eB ± 0.02	4.62eA ± 0.04
	25	0.28dD ± 0.01	0.48dC ± 0.01	0.54dB ± 0.01	0.56dA ± 0.01	4.83dD ± 0.07	5.26dC ± 0.07	8.77dB ± 0.04	11.48dA ± 0.07
	50	0.44cD ± 0.01	0.65cC ± 0.01	0.70cB ± 0.01	0.72cA ± 0.01	6.34cD ± 0.04	7.22cC ± 0.05	11.93cB ± 0.07	13.82cA ± 0.07
	75	0.51bD ± 0.01	0.85bC ± 0.01	0.91bB ± 0.01	0.94bA ± 0.01	8.63bD ± 0.07	12.31bC ± 0.07	13.34bB ± 0.04	17.57bA ± 0.02
	100	0.69aC ± 0.02	0.96aB ± 0.01	1.02aB ± 0.05	1.11aA ± 0.01	9.77aD ± 0.07	14.11aC ± 0.09	18.96aB ± 0.09	22.07aA ± 0.04
*C. iria*	0	0.12eA ± 0.01	0.12eA ± 0.01	0.12eA ± 0.01	0.12eA ± 0.00	2.12eC ± 0.04	2.28eA ± 0.07	2.11eD ± 0.07	2.14eB ± 0.07
	25	0.14dD ± 0.01	0.15dC ± 0.01	0.18dB ± 0.01	0.24dA ± 0.02	2.38dD ± 0.07	3.12dC ± 0.09	3.09dB ± 0.07	3.37dA ± 0.09
	50	0.22cD ± 0.02	0.30cC ± 0.01	0.33cB ± 0.01	0.41cA ± 0.01	3.12cD ± 0.07	4.31cC ± 0.07	4.91cB ± 0.07	6.17cA ± 0.07
	75	0.31bD ± 0.01	0.42bC ± 0.01	0.44bB ± 0.01	0.51bA ± 0.02	4.11bD ± 0.09	6.26bC ± 0.07	6.85bB ± 0.07	8.93bA ± 0.07
	100	0.42aD ± 0.01	0.46aC ± 0.01	0.51aB ± 0.01	0.59aA ± 0.01	5.66aD ± 0.07	7.43aC ± 0.07	7.48aB ± 0.09	9.47aA ± 0.07
*E. hitra*	0	0.21eD ± 0.01	0.24eA ± 0.01	0.23eB ± 0.01	0.22eC ± 0.01	1.63eC ± 0.07	1.84eA ± 0.04	1.61eD ± 0.04	1.64eB ± 0.07
	25	0.31dD ± 0.01	0.36dC ± 0.01	0.64dB ± 0.01	0.68dA ± 0.01	1.80dD ± 0.09	2.80dC ± 0.09	2.85dB ± 0.04	2.93dA ± 0.07
	50	0.43cD ± 0.01	0.57cC ± 0.01	0.71cB ± 0.01	0.77cA ± 0.01	2.66cD ± 0.09	3.32cC ± 0.07	4.26cB ± 0.02	4.34cA ± 0.07
	75	0.57bD ± 0.01	0.81bC ± 0.01	0.84bB ± 0.02	0.85bA ± 0.02	3.94bD ± 0.07	5.65bB ± 0.07	5.31bC ± 0.07	5.67bA ± 0.04
	100	0.64aD ± 0.01	0.88aC ± 0.01	0.96aB ± 0.01	0.98aA ± 0.01	4.45aD ± 0.09	6.45aC ± 0.09	6.80aB ± 0.09	6.98aA ± 0.07
*C. difformis*	0	0.31eA ± 0.01	0.31dA ± 0.01	0.30eB ± 0.01	0.30eB ± 0.01	1.38eC ± 0.04	1.41eB ± 0.04	1.40eB ± 0.04	1.44eA ± 0.04
	25	0.37dD ± 0.01	0.44cC ± 0.01	0.49dB ± 0.01	0.50dA ± 0.01	1.51dD ± 0.07	1.61dC ± 0.04	2.18dB ± 0.07	2.25dA ± 0.07
	50	0.54cC ± 0.01	0.62bB ± 0.01	0.66cA ± 0.01	0.66cA ± 0.00	2.21cC ± 0.07	2.18cD ± 0.05	2.54cB ± 0.07	2.63cA ± 0.07
	75	0.62bD ± 0.01	0.70aC ± 0.01	0.72bB ± 0.01	0.74bA ± 0.01	2.81bD ± 0.09	3.71bC ± 0.04	3.76bB ± 0.07	3.86bA ± 0.07
	100	0.65aD ± 0.01	0.70aC ± 0.01	0.84aB ± 0.01	0.85aA ± 0.01	3.80aC ± 0.04	4.32aB ± 0.02	4.31aB ± 0.04	4.50aA ± 0.07

Data are expressed as means ± standard error. Mean with the same small letters in the column for each concentration and the capital letter within the hours are not significantly different at *p* ≤ 0.05.

**Table 7:** Response of superoxide dismutase, catalase, and peroxidase activity of some crops and weeds due to the foliar spray of *P. hysterophorus* leaf methanolic extract.

Test Plants	Conc. (g L^−1^)	Superoxide Dismutase (Unit g^−1^ FW)				Catalase (µmol g^−1^ FW)				Peroxidase (µmol g^−1^ FW)			
		Hours after Spray				Hours after Spray				Hours after Spray			
		6	24	48	72	6	24	48	72	6	24	48	72
Bambara groundnut	0	2.88eA ± 0.01	2.87eAB ± 0.01	2.83eC ± 0.01	2.86eB ± 0.01	4.03eC ± 0.01	4.07eA ± 0.01	4.03eC ± 0.01	4.05eB ± 0.01	4.88eB ± 0.04	4.86eA ± 0.04	4.87eA ± 0.01	4.83aB ± 0.08
	25	3.10dD ± 0.01	3.74dC ± 0.01	3.88dA ± 0.01	3.76dB ± 0.01	4.52dC ± 0.02	4.78dB ± 0.02	4.85dA ± 0.05	4.78dB ± 0.04	5.92dD ± 0.03	6.54dC ± 0.03	7.72dB ± 0.07	7.46bA ± 0.04
	50	3.83cD ± 0.01	4.43cC ± 0.01	4.66cB ± 0.01	4.68cA ± 0.02	4.83cD ± 0.02	5.19cC ± 0.01	5.33cA ± 0.03	5.25cB ± 0.03	6.47cD ± 0.04	7.50cC ± 0.04	9.07cB ± 0.07	8.85cA ± 0.07
	75	4.57bD ± 0.01	4.87bC ± 0.01	5.00bA ± 0.01	4.98bB ± 0.01	5.23bD ± 0.02	6.02bC ± 0.02	6.40bA ± 0.02	6.11bB ± 0.03	7.51bD ± 0.06	8.82bC ± 0.06	9.92bB ± 0.07	9.65dA ± 0.04
	100	4.76aC ± 0.01	5.32aA ± 0.02	5.21aB ± 0.01	5.20aB ± 0.01	6.04aD ± 0.03	6.77aC ± 0.05	7.72aA ± 0.03	7.61aB ± 0.05	8.70aD ± 0.06	9.65aC ± 0.06	11.21aB ± 0.06	11.06aA ± 0.03
Maize	0	3.11eB ± 0.01	3.14eA ± 0.01	3.10eB ± 0.01	3.07eC ± 0.01	3.85eB ± 0.02	3.80eD ± 0.01	3.84eC ± 0.02	3.88eA ± 0.11	4.47eB ± 0.04	4.46eA ± 0.06	4.42eA ± 0.04	4.41eA ± 0.04
	25	3.74dC ± 0.01	4.11dB ± 0.01	4.22dA ± 0.01	4.11dB ± 0.02	4.28dD ± 0.03	4.36dC ± 0.02	4.68dA ± 0.02	4.54dB ± 0.03	5.14dD ± 0.09	5.52dC ± 0.06	6.56dB ± 0.04	6.25dA ± 0.06
	50	4.21cD ± 0.01	4.64cB ± 0.01	4.82cA ± 0.01	4.62cC ± 0.01	4.69cD ± 0.02	4.90cC ± 0.03	5.81cA ± 0.04	5.77cB ± 0.02	5.99cD ± 0.04	6.21cC ± 0.06	7.58cB ± 0.08	7.35cA ± 0.06
	75	4.73bD ± 0.01	4.99bC ± 0.01	5.30bA ± 0.01	5.17bB ± 0.01	5.21bD ± 0.01	5.85bC ± 0.02	6.44bA ± 0.02	6.36bB ± 0.02	6.51bD ± 0.09	7.61bC ± 0.08	8.55bB ± 0.09	8.36bA ± 0.05
	100	5.03aC ± 0.01	5.54aB ± 0.01	5.65aA ± 0.01	5.55aB ± 0.01	5.72aD ± 0.02	6.30aC ± 0.03	7.27aA ± 0.03	7.18aB ± 0.03	7.61aD ± 0.06	8.57aC ± 0.07	9.40aB ± 0.04	9.13aA ± 0.06
*D. sanguinalis*	0	4.00eA ± 0.01	3.99eA ± 0.01	3.93eB ± 0.01	3.91eC ± 0.01	3.61eC ± 0.01	3.69eA ± 0.02	3.66eB ± 0.02	3.63eBC ± 0.00	5.59eA ± 0.06	5.68eA ± 0.04	5.63eA ± 0.06	5.44eB ± 0.03
	25	4.23dD ± 0.01	4.76dC ± 0.01	5.19dB ± 0.01	5.45dA ± 0.01	4.50dD ± 0.03	5.47dC ± 0.02	5.93dB ± 0.02	7.08dA ± 0.05	7.23dD ± 0.08	8.41dC ± 0.04	9.13dB ± 0.06	10.88dA ± 0.09
	50	4.44cD ± 0.01	5.11cC ± 0.01	5.65cB ± 0.01	6.32cA ± 0.01	5.38cD ± 0.03	6.14cC ± 0.02	7.03cB ± 0.03	7.90cA ± 0.04	8.69cD ± 0.07	9.62cC ± 0.10	10.88cB ± 0.06	12.44cA ± 0.09
	75	4.65bD ± 0.01	5.56bC ± 0.01	5.96bB ± 0.01	7.01bA ± 0.01	6.01bD ± 0.04	7.04bC ± 0.02	7.78bB ± 0.03	9.37bA ± 0.03	10.08bD ± 0.06	11.09bC ± 0.08	12.21bB ± 0.08	14.64bA ± 0.09
	100	4.90aD ± 0.01	5.92aC ± 0.01	6.44aB ± 0.01	7.63aA ± 0.01	6.79aD ± 0.03	7.75aC ± 0.03	9.18aB ± 0.04	10.30aA ± 0.02	11.44aD ± 0.04	12.44aC ± 0.06	14.01aB ± 0.06	16.83aA ± 0.11
*E. indica*	0	3.74eA ± 0.01	3.72eA ± 0.01	3.73eA ± 0.01	3.69eB ± 0.01	2.96eC ± 0.01	3.01eA ± 0.01	2.99eAB ± 0.01	2.96eC ± 0.01	4.91eB ± 0.06	5.15eA ± 0.03	5.00eB ± 0.04	4.98eB ± 0.03
	25	3.89dD ± 0.01	4.12dC ± 0.01	4.31dB ± 0.02	4.66dA ± 0.01	3.63dD ± 0.05	4.01dC ± 0.01	4.66dB ± 0.05	5.51dA ± 0.02	6.26dD ± 0.06	7.97dC ± 0.07	9.09dB ± 0.04	9.44dA ± 0.04
	50	4.09cD ± 0.01	4.79cC ± 0.01	5.03cB ± 0.01	5.12cA ± 0.01	4.45cD ± 0.04	5.15cC ± 0.04	5.70cB ± 0.02	6.63cA ± 0.03	8.03cD ± 0.04	10.38cC ± 0.04	11.05cB ± 0.09	12.25cA ± 0.07
	75	4.32bD ± 0.01	5.01bC ± 0.01	5.76bB ± 0.01	6.65bA ± 0.01	5.35bD ± 0.03	5.74bC ± 0.02	6.84bB ± 0.04	7.79bA ± 0.02	9.20bD ± 0.06	11.47bC ± 0.04	12.10bB ± 0.07	12.87bA ± 0.06
	100	4.66aD ± 0.01	5.44aC ± 0.01	6.01aB ± 0.02	7.13aA ± 0.01	6.12aD ± 0.01	7.07aC ± 0.05	7.80aB ± 0.04	9.14aA ± 0.02	10.07aD ± 0.06	12.58aC ± 0.06	13.51aB ± 0.07	15.54aA ± 0.07
*Ageratum conyzoides*	0	3.21eA ± 0.01	3.20eA ± 0.01	3.20eA ± 0.01	3.15eB ± 0.01	4.40eC ± 0.01	4.44eB ± 0.01	4.48eA ± 0.01	4.42eB ± 0.01	3.36eAB ± 0.04	3.39eA ± 0.01	3.38eB ± 0.01	3.39eAB ± 0.01
	25	3.65dD ± 0.01	3.87dC ± 0.01	3.90dB ± 0.01	3.97dA ± 0.01	5.24dD ± 0.05	5.91dC ± 0.02	6.72dB ± 0.03	7.04dA ± 0.02	4.50dD ± 0.04	5.24dC ± 0.07	5.98dB ± 0.06	6.27dA ± 0.07
	50	3.93cD ± 0.01	4.10cC ± 0.01	4.65cB ± 0.01	5.23cA ± 0.02	5.59cD ± 0.03	6.58cC ± 0.03	7.45cB ± 0.03	7.64cA ± 0.05	5.77cD ± 0.07	6.34cC ± 0.07	7.33cB ± 0.06	7.75cA ± 0.03
	75	4.54bD ± 0.01	4.87bC ± 0.01	5.19bB ± 0.01	5.63bA ± 0.02	6.10bD ± 0.03	7.28bC ± 0.04	7.81bB ± 0.03	9.75bA ± 0.03	6.25bD ± 0.04	7.82bC ± 0.06	8.44bB ± 0.07	9.51bA ± 0.09
	100	4.76aD ± 0.01	5.01aC ± 0.01	5.76aB ± 0.01	6.01aA ± 0.01	7.04aD ± 0.02	7.68aC ± 0.03	9.11aB ± 0.02	11.36aA ± 0.03	7.48aD ± 0.03	9.10aC ± 0.06	10.07aB ± 0.06	10.55aA ± 0.06
*C. iria*	0	4.12eA ± 0.01	4.10eAB ± 0.01	4.09eBC ± 0.01	4.08eC ± 0.01	2.97eD ± 0.01	3.01eC ± 0.01	3.13eA ± 0.01	3.08eB ± 0.02	3.65eC ± 0.03	3.72eA ± 0.06	3.70eAB ± 0.03	3.71eBC ± 0.03
	25	4.67dD ± 0.01	4.95dC ± 0.01	5.41dB ± 0.01	5.53dA ± 0.02	3.37dD ± 0.02	3.67dC ± 0.02	4.20dB ± 0.03	4.93dA ± 0.03	4.97dD ± 0.04	5.48dC ± 0.08	6.00dB ± 0.06	6.53dA ± 0.07
	50	4.98cD ± 0.01	5.34cC ± 0.01	5.81cB ± 0.02	6.45cA ± 0.02	3.82cD ± 0.01	4.45cC ± 0.03	5.02cB ± 0.05	5.72cA ± 0.02	5.56cC ± 0.07	5.99cB ± 0.10	7.92cA ± 0.11	8.04cA ± 0.06
	75	5.22bD ± 0.02	5.69bC ± 0.01	6.32bB ± 0.01	6.77bA ± 0.01	4.73bD ± 0.04	5.32bC ± 0.02	5.91bB ± 0.02	7.03bA ± 0.05	6.51bD ± 0.08	7.25bC ± 0.06	9.11bB ± 0.06	9.40bA ± 0.04
	100	5.43aD ± 0.01	5.99aC ± 0.01	6.88aB ± 0.01	7.21aA ± 0.01	5.14aD ± 0.03	5.81aC ± 0.03	7.21aB ± 0.04	7.81aA ± 0.04	7.64aD ± 0.08	8.76aC ± 0.06	10.50aB ± 0.06	10.85aA ± 0.04
*E. hitra*	0	2.54eAB ± 0.01	2.56eA ± 0.01	2.52eB ± 0.02	2.52eB ± 0.02	2.87eA ± 0.01	2.84eB ± 0.01	2.87eA ± 0.02	2.87eA ± 0.01	3.46eBC ± 0.01	3.51eB ± 0.06	3.50eA ± 0.03	3.51eC ± 0.03
	25	2.66dD ± 0.01	2.86dC ± 0.01	2.95dB ± 0.02	3.19dA ± 0.01	3.73dD ± 0.03	4.00dC ± 0.02	4.60dB ± 0.05	5.34dA ± 0.02	4.12dC ± 0.06	5.23dB ± 0.09	5.36dA ± 0.05	5.51dA ± 0.11
	50	2.87cD ± 0.01	3.09cC ± 0.01	3.46cB ± 0.01	3.75cA ± 0.01	4.42cD ± 0.02	5.06cC ± 0.03	5.52cB ± 0.03	5.91cA ± 0.03	5.17cD ± 0.04	6.58cC ± 0.06	7.67cB ± 0.10	8.03cA ± 0.08
	75	3.43bD ± 0.01	3.85bC ± 0.01	4.09bB ± 0.01	4.42bA ± 0.02	5.47bD ± 0.03	5.72bC ± 0.02	6.16bB ± 0.02	7.14bA ± 0.03	6.28bD ± 0.06	8.37bC ± 0.11	8.67bB ± 0.11	8.78bA ± 0.07
	100	3.71aD ± 0.01	4.04aC ± 0.01	4.47aB ± 0.01	4.78aA ± 0.02	5.74aD ± 0.02	6.49aC ± 0.02	7.39aB ± 0.03	7.57aA ± 0.03	7.51aD ± 0.06	9.46aC ± 0.06	10.31aB ± 0.07	10.58aA ± 0.09
*C. difformis*	0	2.77eAB ± 0.01	2.73eC ± 0.01	2.75eBC ± 0.01	2.78eA ± 0.01	2.57eC ± 0.02	2.59eB ± 0.03	2.60eA ± 0.03	2.59eB ± 0.01	2.40eA ± 0.03	2.44eA ± 0.06	2.43eA ± 0.07	2.42eA ± 0.09
	25	3.09dD ± 0.01	3.66dC ± 0.01	3.87dA ± 0.01	3.85dB ± 0.02	2.94dD ± 0.05	2.99dB ± 0.02	3.01dA ± 0.01	2.95dC ± 0.03	2.56dD ± 0.04	2.69dC ± 0.08	2.75dB ± 0.10	2.66dA ± 0.07
	50	3.54cD ± 0.02	4.10cC ± 0.01	4.63cB ± 0.03	4.65cA ± 0.02	3.54cD ± 0.02	3.66cC ± 0.04	3.93cA ± 0.03	3.84cB ± 0.02	2.80cD ± 0.04	3.18cC ± 0.09	3.43cB ± 0.06	3.47cA ± 0.11
	75	4.01bD ± 0.01	4.54bC ± 0.01	4.87bB ± 0.01	4.91bA ± 0.02	3.71bD ± 0.02	4.13bC ± 0.04	4.54bA ± 0.03	4.46bB ± 0.02	3.03bD ± 0.06	4.13bC ± 0.04	4.28bB ± 0.04	4.28bA ± 0.09
	100	4.22aD ± 0.01	4.75aC ± 0.01	4.92aB ± 0.01	4.95aA ± 0.01	4.42aD ± 0.03	5.03aC ± 0.01	5.24aA ± 0.02	5.13aB ± 0.03	3.67aD ± 0.04	4.51aC ± 0.08	4.68aB ± 0.04	4.71aA ± 0.09

Data are expressed as means ± standard error. Mean with the same small letters in the column for each concentration and the capital letter within the hours are not significantly different at *p* ≤ 0.05.

## Data Availability

Not applicable.

## Supplementary Materials

The following supporting information can be downloaded at: https://www.mdpi.com/article/10.3390/plants11233209/s1. Table S1. Percent reduction in chlorophyll-ɑ and chlorophyll-ɓ content to a foliar spray of *P. hysterophorus* leaf extract on some crops and weeds, compared with control; Table S2. Percent reduction in total chlorophyll content and carotenoids to a foliar spray of *P. hysterophorus* leaf extract on some crops and weeds, compared with control; Table S3. Percent reduction in photosynthesis rate, stomatal conductance, and transpiration rate to a foliar spray of *P. hysterophorus* leaf extract on some crops and weeds, compared with control; Table S4. Response of malondialdehyde and proline content to a foliar spray of *P. hysterophorus* leaf extract on some crops and weeds; Table S5. Response of superoxide dismutase, catalase, and peroxidase activity to a foliar spray of *P. hysterophorus* leaf extract on some crops and weeds.
