# Supplementary material for: Determination and Quantification of Phytochemicals from the Leaf Extract of Parthenium hysterophorus L. and Their Physio-Biochemical Responses to Several Crop and Weed Species
Source: Plants (Basel). 2022 Nov 23;11(23):3209. doi: 10.3390/plants11233209 (PMC9736957; doi:10.3390/plants11233209)
Supplement: Supplementary file 1 [file plants-11-03209-s001.zip › plants-1943700-supplementary.pdf]

Table S1. Percent reduction of chlorophyll-ɑ and chlorophyll-ɓ content to a foliar spray of *P. hysterophorus* leaf extract of some crops and weeds comparison with control.

Test Plants	Concentration (g L^{-1})	Chlorophyll-ɑ (mg g^{-1} FW)				Chlorophyll-ɓ (mg g^{-1} FW)			
		Hours after Spray				Hours after Spray			
		6	24	48	72	6	24	48	72
Bambara groundnut	0	0	0	0	0	0	0	0	0
	25	0	3.16	1.97	3.41	0.25	2.87	1.57	0.51
	50	8.64	7.59	8.22	5.11	13.04	14.09	14.21	9.48
	75	15.43	9.81	14.14	12.28	15.60	18.27	19.73	12.05
	100	18.51	14.55	25.32	25.25	17.64	21.93	22.63	15.89
Maize	0	0	0	0	0	0	0	0	0
	25	6.75	1.90	3.65	1.16	1.82	3.92	5.87	3.27
	50	7.07	7.63	6.50	6.20	3.44	7.64	10.12	7.58
	75	8.68	11.06	9.34	8.91	3.84	10.33	16.39	11.06
	100	18.32	12.97	13.82	12.01	10.12	14.46	18.82	12.29
D. sanguinalis	0	0	0	0	0	0	0	0	0
	25	9.43	8.55	4.33	5.92	4.99	9.43	10.36	21.15
	50	15.72	12.82	28.74	33.59	12.65	30.07	30.22	40.94
	75	24.84	22.36	49.60	50.98	40.28	48.04	54.13	66.15
	100	36.79	52.96	61.02	79.84	56.50	70.64	73.81	81.55
E. indica	0	0	0	0	0	0	0	0	0
	25	3.22	18.09	17.43	30.82	19.12	20.21	24.05	37.47
	50	13.54	20.06	27.75	62.72	23.50	32.91	36.08	42.41
	75	18.06	35.19	56.93	69.53	31.75	35.42	48.47	60.47
	100	33.22	57.56	66.54	78.85	51.05	51.87	72.17	80.91
Ageratum conyzoides	0	0	0	0	0	0	0	0	0
	25	10.29	46.62	46.64	56.14	5.20	19.10	17.44	30.00
	50	32.89	51.35	55.12	61.75	22.91	21.34	22.09	39.00
	75	40.53	55.40	60.42	65.96	42.70	38.20	43.02	55.00
	100	43.18	58.78	63.60	71.22	48.95	49.43	54.65	74.00
iria	0	0	0	0	0	0	0	0	0
	25	1.82	0.65	0.39	11.33	28.61	36.33	46.29	51.97
	50	3.04	3.25	4.74	24.00	48.50	50.54	52.68	59.10
	75	8.23	7.81	15.81	65.33	52.58	58.19	61.89	62.53
	100	24.69	28.33	58.89	72.00	52.58	62.56	66.49	68.33
E. hitra	0	0	0	0	0	0	0	0	0
	25	130.08	131.48	130.20	107.57	122.90	119.39	118.37	116.14
	50	130.89	135.18	132.29	115.90	129.95	128.87	125.64	123.31

Test Plants	Concentration (g L^{-1})	Chlorophyll-ɑ (mg g^{-1} FW)				Chlorophyll-ϐ (mg g^{-1} FW)			
		Hours after Spray				Hours after Spray			
		**6**	**24**	**48**	**72**	**6**	**24**	**48**	**72**
	75	133.33	139.81	145.83	120.45	137.00	130.17	126.49	124.66
	100	152.03	156.48	160.41	125.00	139.64	135.34	129.91	130.94
C. difformis	0	0	0	0	0	0	0	0	0
	25	8.33	6.05	1.84	1.31	0.34	2.93	1.74	1.39
	50	10.41	5.73	2.95	2.63	3.84	8.79	9.09	8.74
	75	10.71	11.78	7.01	7.23	8.74	12.82	15.38	15.03
	100	13.98	21.33	27.67	17.43	13.28	17.58	25.87	23.07

Table S2. Percent reduction of total chlorophyll content and carotenoids to a foliar spray of *P. hysterophorus* leaf extract of some crops and weeds compare with control.

Test Plants	Concentration ($g\ L^{-1}$)	Total Chlorophyll ($mg\ g^{-1}$ FW)				Carotenoids ($mg\ g^{-1}$ FW)			
		Hours after Spray				Hours after Spray			
		6	24	48	72	6	24	48	72
Bambara Groundnut	0	0	0	0	0	0	0	0	0
	25	0.13	3.00	1.75	1.75	1.28	3.22	5.00	5.16
	50	11.04	11.15	11.54	7.61	7.05	10.96	12.14	12.90
	75	15.52	14.44	17.25	12.15	10.89	14.83	17.85	17.41
	100	18.04	18.59	23.84	19.91	15.38	20.64	20.71	20.64
Maize	0	0	0	0	0	0	0	0	0
	25	3.72	3.21	5.13	2.54	2.95	3.59	5.52	2.95
	50	4.84	7.64	8.91	7.10	5.32	7.18	11.65	8.28
	75	5.71	10.58	14.05	10.32	10.05	14.97	17.79	13.60
	100	13.29	13.94	17.16	12.19	13.60	19.76	20.85	18.34
D. sanguinalis	0	0	0	0	0	0	0	0	0
	25	6.70	9.22	8.50	16.68	16.18	17.64	21.05	22.67
	50	13.86	24.10	31.34	37.27	28.90	31.17	40.93	45.34
	75	35.11	38.63	52.61	61.53	43.35	50.00	70.76	72.67
	100	49.43	64.47	69.86	81.06	53.75	68.82	77.19	93.60
E. indica	0	0	0	0	0	0	0	0	0
	25	15.11	17.61	21.71	35.33	0.78	5.6	3.27	19.04
	50	19.88	28.50	35.44	46.88	13.38	17.6	23.77	35.71
	75	26.93	35.22	51.31	63.27	30.70	37.6	50.81	62.69
	100	45.90	52.60	70.16	80.25	34.64	59.2	61.47	75.39
Ageratum conyzoides	0	0	0	0	0	0	0	0	0
	25	14.69	34.27	40.48	50.00	9.40	25.00	31.03	35.89
	50	29.63	43.81	46.91	52.84	22.22	37.93	43.96	47.86
	75	39.43	52.06	56.56	62.95	35.04	37.93	44.82	52.99
	100	45.61	55.92	60.05	71.76	41.02	53.44	63.79	70.08
C. iria	0	0	0	0	0	0	0	0	0
	25	15.97	19.91	28.26	33.87	3.12	17.72	24.67	36.25
	50	27.05	28.97	33.85	43.59	23.75	25.94	29.22	40.00
	75	31.65	37.59	46.42	62.59	36.87	41.13	56.49	69.37
	100	39.42	44.42	61.64	69.80	42.50	55.06	68.83	73.12
E. hitra	0	0	0	0	0	0	0	0	0
	25	123.07	123.46	123.93	112.92	111.39	115.38	106.66	103.75
	50	130.48	130.79	127.87	120.50	113.92	114.10	108.00	106.25

Test Plants	Concentration (g L^{-1})	Total Chlorophyll (mg g^{-1} FW)				Carotenoids (mg g^{-1} FW)			
		Hours after Spray				Hours after Spray			
		6	24	48	72	6	24	48	72
	75	135.89	133.43	132.42	123.31	116.45	110.25	110.66	110.00
	100	143.87	142.22	138.78	128.93	117.72	108.97	114.66	110.00
C. difformis	0	0	0	0	0	0	0	0	0
	25	4.66	4.59	1.79	1.35	3.04	6.96	7.45	3.06
	50	7.39	7.15	6.10	5.59	7.31	12.65	13.04	11.04
	75	9.80	12.26	11.31	11.01	12.80	19.62	19.87	15.95
	100	13.66	19.59	26.75	20.16	15.85	25.31	29.81	23.31

Table S3. Percent reduction of photosynthesis rate, stomatal conductance and transpiration rate to a foliar spray of *P. hysterophorus* leaf extract of some crops and weeds comparison with control.

Test Plants	Con. (g L^{-1})	Photosynthesis Rate (μmol m^{-2} s^{-1})				Stomatal Conductance (mol m^{-2} s^{-1})				Transpiration Rate (mmol m^{-2} s^{-1})			
		Hours after Spray				Hours after Spray				Hours after Spray			
		6	24	48	72	6	24	48	72	6	24	48	72
Bambara groundnut	0	0	0	0	0	0	0	0	0	0	0	0	0
	25	1.89	2.19	6.77	5.50	5.55	11.11	11.11	11.11	5.43	7.17	9.26	9.32
	50	5.50	14.04	15.63	14.58	11.11	16.67	22.22	21.67	11.18	12.53	13.35	12.71
	75	11.85	17.62	25.09	21.83	16.16	27.77	33.33	31.11	17.71	19.70	21.66	20.63
	100	18.02	29.58	30.17	26.76	27.78	38.89	44.44	42.77	23.91	25.33	29.90	29.84
Maize	0	0	0	0	0	0	0	0	0	0	0	0	0
	25	0.38	4.83	6.57	5.03	8.00	12.00	12.00	11.53	3.79	4.08	6.31	5.99
	50	3.76	11.15	13.35	12.45	12.00	16.00	24.00	23.07	7.40	7.96	9.61	9.21
	75	10.35	24.10	25.13	24.36	16.00	20.00	28.00	27.69	16.57	20.07	21.85	21.13
	100	14.65	28.90	31.20	26.08	24.00	28.00	36.00	35.76	21.88	27.18	32.65	32.19
D. sanguinalis	0	0	0	0	0	0	0	0	0	0	0	0	0
	25	15.70	15.06	22.15	28.22	6.12	18.75	37.50	38.77	19.61	21.22	26.64	26.73
	50	30.12	32.53	38.73	48.47	59.18	64.58	75.00	75.51	22.77	28.34	33.31	33.33
	75	51.14	54.57	73.28	86.04	108.16	106.25	104.16	102.04	48.38	68.09	77.96	78.01
	100	75.18	83.73	95.38	98.71	120.40	116.66	112.50	110.20	69.91	124.58	113.32	113.33
E. indica	0	0	0	0	0	0	0	0	0	0	0	0	0
	25	19.21	14.99	27.64	33.63	21.21	39.39	48.48	46.87	9.71	16.90	25.24	25.26
	50	37.06	27.81	37.70	65.06	36.36	66.66	72.72	71.87	24.10	37.03	42.15	42.26
	75	62.63	62.53	87.20	92.52	60.60	93.93	96.96	96.87	51.64	61.97	74.75	74.80
	100	83.37	92.19	98.49	99.82	87.87	115.15	112.12	112.50	67.32	125.55	123.87	123.81
Ageratum	0	0	0	0	0	0	0	0	0	0	0	0	0

Test Plants	Con. (g L^{-1})	Photosynthesis Rate (µmol m^{-2} s^{-1})				Stomatal Conductance (mol m^{-2} s^{-1})				Transpiration Rate (mmol m^{-2} s^{-1})			
		Hours after Spray				Hours after Spray				Hours after Spray			
		6	24	48	72	6	24	48	72	6	24	48	72
conyzoides	25	10.96	23.35	2.96	13.77	2.56	23.68	36.84	38.46	10.98	12.36	16.21	16.10
	50	30.32	38.02	34.09	34.15	5.12	50.00	60.52	64.10	19.13	43.51	61.50	61.58
	75	45.32	54.26	59.18	61.12	23.07	63.15	68.42	71.79	30.00	72.43	72.64	72.86
	100	58.58	75.52	66.89	70.27	76.92	81.57	86.84	87.17	47.65	84.54	84.77	84.75
C. iria	0	0	0	0	0	0	0	0	0	0	0	0	0
	25	5.74)	11.26	16.10	17.25	2.50	4.87	7.69	7.5	8.58	17.59	23.97	20.90
	50	21.28	24.30	29.83	30.84	10.00	14.63	17.94	20.00	18.80	26.09	29.76	26.41
	75	40.99	46.01	49.96	54.81	50.00	60.97	64.10	65.00	28.60	32.89	43.33	40.35
	100	51.71	60.93	72.00	71.96	62.50	82.92	87.17	87.50	36.97	49.04	61.60	59.72
E. hitra	0	0	0	0	0	0	0	0	0	0	0	0	0
	25	15.87	23.82	31.20	31.18	12.00	16.66	20.83	20.83	5.68	27.95	33.24	33.56
	50	31.68	40.52	53.80	55.25	32.00	37.50	45.83	45.83	9.15	42.83	44.78	44.70
	75	51.93	54.35	64.81	66.08	44.00	54.16	58.33	58.33	20.11	55.21)	65.92	66.15
	100	61.54	73.46	74.42	75.35	52.00	62.50	62.50	66.66	61.58	83.44	94.57	94.56
C. difformis	0	0	0	0	0	0	0	0	0	0	0	0	0
	25	1.20	4.15	6.36	5.36	0	5.88	5.88	5.88	7.40	8.13	14.15	14.76
	50	6.56	8.39	12.44	11.95	11.76	11.76	17.64	17.64	10.34	8.87	19.15	19.30
	75	15.70	18.01	20.09	19.64	11.76	17.64	29.41	23.52	19.54	14.80	24.23	24.11
	100	22.60	24.41	27.97	26.11	17.64	23.52	29.41	23.52	27.41	28.88	33.37	33.33

Table S4. Response of malondialdehyde and proline content to a foliar spray of *P. hysterophorus* leaf extract of some crops and weeds.

Test Plants	Concentration (g L^{-1})	Malondialdehyde Content (µmol g^{-1} FW)				Proline Content (µmol g^{-1} FW)			
		Hours after Spray				Hours after Spray			
		6	24	48	72	6	24	48	72
Bambara Groundnut	0	0	0	0	0	0	0	0	0
	25	7.5	14.634146	26.3157895	26.315789	6.1797753	14.20455	42.372881	40.8046
	50	20	39.02439	55.2631579	55.263158	19.101124	50.56818	85.310734	78.73563
	75	47.5	56.097561	94.7368421	97.368421	46.067416	78.97727	123.16384	118.9655
	100	77.5	97.560976	131.578947	134.21053	69.101124	146.5909	190.39548	193.6782
Maize	0	0	0	0	0	0	0	0	0
	25	14.285714	28.571429	42.8571429	41.666667	7.1428571	23.3871	29.508197	25
	50	28.571429	42.857143	71.4285714	66.666667	23.809524	46.77419	78.688525	78.22581
	75	42.857143	71.428571	85.7142857	83.333333	41.269841	74.19355	124.59016	125
	100	57.142857	85.714286	114.285714	116.66667	67.460317	140.3226	182.78689	182.2581
D. sanguinalis	0	0	0	0	0	0	0	0	0

Test Plants	Concentration (g L^{-1})	Malondialdehyde Content (μmol g^{-1} FW)				Proline Content (μmol g^{-1} FW)			
		Hours after Spray				Hours after Spray			
		**6**	**24**	**48**	**72**	**6**	**24**	**48**	**72**
	25	17.948718	60.97561	123.529412	148.48485	22.262774	62.35294	67.322835	210.3586
	50	56.410256	73.170732	167.647059	242.42424	41.605839	130.5882	210.62992	293.6255
	75	120.51282	156.09756	267.647059	290.90909	95.985401	203.9216	329.52756	372.51
	100	194.87179	246.34146	355.882353	409.09091	130.29197	241.5686	374.80315	478.8845
E. indica	0	0	0	0	0	0	0	0	0
	25	70.37037	144.44444	200	272	13	30.73394	117.28972	131.2796
	50	107.40741	188.88889	259.259259	412	74	94.0367	237.85047	313.2701
	75	244.44444	274.07407	403.703704	516	107.5	161.9266	292.52336	411.8483
	100	285.18519	459.25926	496.296296	596	178.5	217.4312	386.91589	492.891
Ageratum conyzoides	0	0	0	0	0	0	0	0	0
	25	21.73913	108.69565	145.454545	154.54545	12.850467	15.6044	90.652174	148.4848
	50	91.304348	182.6087	218.181818	227.27273	48.130841	58.68132	159.34783	199.1342
	75	121.73913	269.56522	313.636364	327.27273	101.63551	170.5495	190	280.303
	100	200	317.3913	363.636364	404.54545	128.27103	210.1099	312.17391	377.7056
C. iria	0	0	0	0	0	0	0	0	0
	25	16.666667	25	50	100	12.264151	36.84211	46.445498	57.47664
	50	83.333333	150	175	241.66667	47.169811	89.03509	132.70142	188.3178
	75	158.33333	250	266.666667	325	93.867925	174.5614	224.64455	317.2897
	100	250	283.33333	325	391.66667	166.98113	225.8772	254.50237	342.5234
E. hitra	0	0	0	0	0	0	0	0	0
	25	47.619048	50	178.26087	209.09091	10.429448	52.17391	77.018634	78.65854
	50	104.7619	137.5	208.695652	250	63.190184	80.43478	164.59627	164.6341
	75	171.42857	237.5	265.217391	286.36364	141.71779	207.0652	229.81366	245.7317
	100	204.7619	266.66667	317.391304	345.45455	173.00613	250.5435	322.36025	325.6098
C. difformis	0	0	0	0	0	0	0	0	0
	25	19.354839	41.935484	63.3333333	66.666667	9.4202899	14.1844	55.714286	56.25
	50	74.193548	100	120	120	60.144928	54.60993	81.428571	82.63889
	75	100	125.80645	140	146.66667	103.62319	163.1206	168.57143	168.0556
	100	109.67742	125.80645	180	183.33333	175.36232	206.383	207.85714	212.5

Table S5. Response of superoxide dismutase, catalase, and peroxidase activity to a foliar spray of *P. hysterophorus* leaf extract of some crops and weeds.

**Test Plants**	**Con. (g L^{-1})**	**Superoxide Dismutase (Unit g^{-1} FW)**				**Catalase (µmol g^{-1} FW)**				**Peroxidase (µmol g^{-1} FW)**			
		**Hours after Spray**				**Hours after Spray**				**Hours after Spray**			
		**6**	**24**	**48**	**72**	**6**	**24**	**48**	**72**	**6**	**24**	**48**	72
Bambara Groundnut	0	0	0	0	0	0	0	0	0	0	0	0	0
	25	7.6388889	30.313589	37.102473	31.468531	12.158809	17.444717	20.347395	18.024691	21.311475	34.567901	58.521561	54.451346
	50	32.986111	54.355401	64.664311	63.636364	19.851117	27.518428	32.258065	29.62963	32.581967	54.320988	86.2423	83.229814
	75	58.680556	69.686411	76.678445	74.125874	29.776675	47.911548	58.808933	50.864198	53.893443	81.481481	103.6961	99.792961
	100	65.277778	85.365854	84.09894	81.818182	49.875931	66.339066	91.563275	87.901235	78.278689	98.559671	130.1848	128.98551
Maize	0	0	0	0	0	0	0	0	0	0	0	0	0
	25	20.257235	30.89172	36.129032	33.876221	11.168831	14.736842	21.875	17.010309	14.988814	23.766816	48.41629	41.723356
	50	35.369775	47.770701	55.483871	50.488599	21.818182	28.947368	51.302083	48.71134	34.004474	39.237668	71.493213	66.666667
	75	52.090032	58.917197	70.967742	68.403909	35.324675	53.947368	67.708333	63.917526	45.637584	70.627803	93.438914	89.569161
	100	61.736334	76.433121	82.258065	80.781759	48.571429	65.789474	89.322917	85.051546	70.246085	92.152466	112.66968	107.02948
D. sanguinalis	0	0	0	0	0	0	0	0	0	0	0	0	0
	25	5.75	19.298246	32.061069	39.386189	24.65374	48.238482	62.021858	95.041322	29.338104	48.06338	62.166963	100
	50	11	28.070175	43.765903	61.636829	49.030471	66.395664	92.076503	117.63085	55.456172	69.366197	93.250444	128.67647
	75	16.25	39.348371	51.653944	79.283887	66.204986	90.785908	112.56831	158.12672	80.322004	95.246479	116.87389	169.11765
	100	22.5	48.370927	63.867684	95.140665	88.088643	110.0271	150.81967	183.74656	104.65116	119.01408	148.84547	209.375
E. indica	0	0	0	0	0	0	0	0	0	0	0	0	0
	25	4.0106952	10.752688	15.549598	26.287263	22.635135	33.222591	55.852843	86.148649	27.494908	54.757282	81.8	89.558233
	50	9.3582888	28.763441	34.852547	38.753388	50.337838	71.096346	90.635452	123.98649	63.543788	101.5534	121	145.98394
	75	15.508021	34.677419	54.423592	80.216802	80.743243	90.697674	128.76254	163.17568	87.372709	122.71845	142	158.43373
	100	24.59893	46.236559	61.126005	93.224932	106.75676	134.88372	160.86957	208.78378	105.09165	144.27184	170.2	212.04819
Ageratum conyzoides	0	0	0	0	0	0	0	0	0	0	0	0	0
	25	13.707165	20.9375	21.875	26.031746	19.090909	33.108108	50	59.276018	33.928571	54.572271	76.923077	84.955752
	50	22.429907	28.125	45.3125	66.031746	27.045455	48.198198	66.294643	72.850679	71.72619	87.020649	116.86391	128.61357
	75	41.433022	52.1875	62.1875	78.730159	38.636364	63.963964	74.330357	120.58824	86.011905	130.67847	149.70414	180.53097
	100	48.286604	56.5625	80	90.793651	60	72.972973	103.34821	157.01357	122.61905	168.43658	197.92899	211.20944
C. iria	0	0	0	0	0	0	0	0	0	0	0	0	0
	25	13.349515	20.731707	32.273839	35.539216	13.468013	21.92691	34.185304	60.064935	36.164384	47.311828	62.162162	76.010782
	50	20.873786	30.243902	42.05379	58.088235	28.619529	47.840532	60.383387	85.714286	52.328767	61.021505	114.05405	116.71159
	75	26.699029	38.780488	54.523227	65.931373	59.259259	76.744186	88.817891	128.24675	78.356164	94.892473	146.21622	153.36927
	100	31.796117	46.097561	68.215159	76.715686	73.063973	93.023256	130.35144	153.57143	109.31507	135.48387	183.78378	192.45283
E. hitra	0	0	0	0	0	0	0	0	0	0	0	0	0
	25	4.7244094	11.71875	17.063492	26.587302	29.965157	40.84507	60.278746	86.062718	19.075145	49.002849	53.142857	56.980057
	50	12.992126	20.703125	37.301587	48.809524	54.006969	78.169014	92.334495	105.92334	49.421965	87.464387	119.14286	128.77493
	75	35.03937	50.390625	62.301587	75.396825	90.592334	101.40845	114.63415	148.78049	81.50289	138.46154	147.71429	150.14245
	100	46.062992	57.8125	77.380952	89.68254	100	128.52113	157.49129	163.76307	117.05202	169.51567	194.57143	201.4245

Test Plants	Con. (g L^{-1})	Superoxide Dismutase (Unit g^{-1} FW)				Catalase (μmol g^{-1} FW)				Peroxidase (μmol g^{-1} FW)			
		Hours after Spray				Hours after Spray				Hours after Spray			
		**6**	**24**	**48**	**72**	**6**	**24**	**48**	**72**	**6**	**24**	**48**	72
C. difformis	0	0	0	0	0	0	0	0	0	0	0	0	0
	25	11.552347	34.065934	40.727273	38.489209	14.396887	15.444015	15.769231	13.899614	6.6666667	10.245902	13.168724	9.9173554
	50	27.797834	50.18315	68.363636	67.266187	37.743191	41.312741	51.153846	48.262548	16.666667	30.327869	41.152263	43.38843
	75	44.765343	66.300366	77.090909	76.618705	44.357977	59.459459	74.615385	72.200772	26.25	69.262295	76.131687	76.859504
	100	52.34657	73.992674	78.909091	78.057554	71.984436	94.208494	101.53846	98.069498	52.916667	84.836066	92.592593	94.628099
